# Statistical shape refinement and genetic algorithm calibration of design response spectra based on strong-motion records

**DOI:** 10.1371/journal.pone.0348599

**Published:** 2026-07-01

**Authors:** Xin Han, Chaoyu Chang, Jingshan Bo, Shun Yang, Qiangguo Song, Shaopeng Wang, Mei Guo

**Affiliations:** 1 Gansu Earthquake Agency Lanzhou Institute of Seismolog, CEA, Lanzhou, Gansu, China; 2 Institute of Disaster Prevention, Sanhe, Hebei, China; 3 Earthquake Agency of Ningxia Hui Autonomous Region, Yinchuan, Ningxia, China; University of Catania, ITALY

## Abstract

Ground-motion parameters for different site conditions are conventionally prescribed in seismic design codes worldwide, based on post-earthquake damage surveys and statistical analyses of strong-motion databases. Design response spectra thus constitute the primary basis for engineering aseismic design, yet their shapes and characteristic parameters differ markedly among national codes. Systematic computations reveal that code-specified spectral shapes deviate appreciably from recorded earthquake spectra, providing an incomplete representation of site-specific ground-motion characteristics.To address this limitation, 1227 horizontal acceleration records were compiled and classified by magnitude, source distance and site class. Mean dynamic amplification spectra were computed for each subset, and numerical computation were performed to derive an improved analytical expression for the design spectrum. A genetic-algorithm-based calibration procedure was subsequently developed to determine optimal model parameters. The applicability of the proposed approach was examined by calibrating spectra for four independent strong-motion records; spectral parameters obtained with the improved shape were compared with those derived from the conventional code spectrum.Results demonstrate that the refined spectral form captures the frequency-dependent characteristics of earthquake response spectra more accurately than existing representations. The proposed calibration framework offers a valuable reference for advancing design-response-spectrum studies and for the potential updating of seismic design codes.

## 1. Introduction

The design response spectrum underpins seismic-resistant structural design; its fidelity in reproducing the frequency-dependent amplitude characteristics of earthquake ground motion is therefore both a fundamental requirement and a non-negotiable criterion.To construct the design spectrum, an extensive strong-motion database is first scrutinized to assemble sets of records with comparable site, source, and path attributes. For each set, acceleration response spectra at a prescribed damping ratio are computed and normalized by the peak ground acceleration (PGA) of the corresponding records. The normalized spectra are then subjected to statistical analysis and smoothing, followed by adjustments that account for prevailing economic constraints and performance objectives. The resulting spectrum provides a design-compatible curve for evaluating the seismic demand that buildings may experience during their design service life.

**Fig 1:**
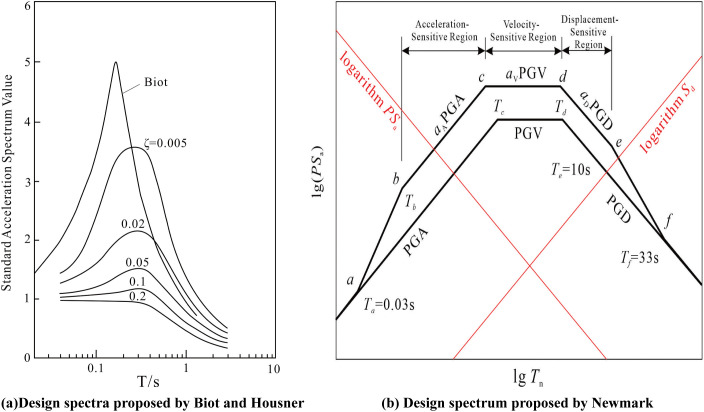
Comparison of proposed design response spectra.

Response spectrum theory originated in the 1930s, when M. A. Biot first defined the earthquake response spectrum for a single-degree-of-freedom (SDOF) system within the framework of elastic vibration theory [1,2]. The world’s first strong-motion accelerograph was developed in the United States in 1932, providing the inaugural set of high-quality strong-motion records. Leveraging this database and Biot’s formulation, Housner G.W. employed analog-circuit simulations to compute spectral curves for major U.S. earthquakes, introduced the concept of the seismic design response spectrum with a smoothed shape, and demonstrated the pronounced influence of damping on strong ground motion. His curve-based design spectrum was subsequently incorporated into the California seismic design code in the 1950s [3,4]. In the 1960s, N. M. Newmark proposed replacing the smooth curve with a piece-wise linear representation and advanced a three-parameter calibration method to further simplify the spectrum [5,6]. Fig 1 compares the design-spectrum curves proposed by Biot, Housner, and N. M. Newmark [7] (Fig 1). Following the work of Newmark and Hall, seismic design codes worldwide have widely adopted this simplified framework, using a horizontal plateau to characterize the medium-frequency spectral shape [8–11]. Major international standards, including Eurocode EN 1998–1, ASCE/SEI 7–10 in the United States, the Turkish Building Seismic Design Code, and the Iranian Code of Practice for Seismic Resistant Design of Buildings, all employ a flat-segment design spectrum. Fundamentally, these are simplified piecewise linear spectra based on statistical averaging. However, this globally prevalent assumption of a horizontal plateau overlooks the pronounced influence of site conditions on response spectrum characteristics and fails to accurately capture the systematic variations in ground motion parameters across different site categories [12–16].

Extensive research has demonstrated that site conditions constitute a critical factor governing ground motion parameters. Guo et al. [12] identified through statistical analysis that site conditions exert a significant influence on the maximum values of design response spectra. Lü and colleagues [13] proposed recommended values for site influence coefficients based on numerical analyses of site-specific models using data from representative regions, systematically examining the critical issues concerning the impact of site conditions on ground motion parameters. Bo et al. [14] concluded that soil layer structures significantly affect response spectrum characteristic parameters; specifically, characteristic periods increase with greater overburden thickness. Zhou et al. [15] conducted statistical analyses of the combined effects of magnitude, epicentral distance, and site conditions on response spectrum characteristics, concluding that buildings designed according to code provisions generally withstand seismic actions exceeding the code-specified levels. Consequently, the range of Site Class II was deliberately expanded as a negotiated compromise. Furthermore, although ground motion selection tools developed by Wang et al. [16] provide extensive record libraries, they remain constrained by fixed spectral shapes prescribed in design codes for record matching, thereby failing to fundamentally address the systematic discrepancies between code-specified spectra and actual earthquake response spectra. These studies collectively indicate that site dynamic properties play a decisive role in shaping response spectrum morphology; nevertheless, current code spectra continue to employ uniform piecewise linear forms without adequately accounting for spectral shape variations induced by site classification.

At present, the calibration of site-specific response spectra in most countries still follows the standard shape prescribed by national seismic design codes, wherein the mid-period range is conventionally represented by a horizontal straight line to characterize its spectral properties [8–11]. The overall profile of a design spectrum is governed by the selected ground-motion parameters. For critical or special facilities, these parameters are usually established directly through near-fault ground-motion simulation, probabilistic seismic hazard analysis, or careful selection of recorded accelerograms [17–24]; ordinary projects simply adopt the codified spectrum assigned to the corresponding site class. Code spectra are obtained by statistically averaging and smoothing normalized acceleration response spectra computed from strong-motion recordings observed on diverse site conditions [25,26]. Nevertheless, response spectra from individual earthquakes exhibit highly complex shapes; the aforementioned averaging process can only supply a crude intensity estimate over the mid-period band and fails to capture the genuine variability of ground-motion parameters introduced by differences in tectonic setting, local site conditions, and source characteristics [12–16]. Moreover, the assumption of a frequency-independent horizontal segment introduces a systematic bias that amplifies the scatter between the design spectrum and actual spectra, while obscuring the dominant influence of site dynamic properties on ground-motion parameters.

Notably, seismic resilience (SR) has emerged in recent years as a central paradigm in earthquake engineering for evaluating structural capabilities to withstand, recover from, and adapt to seismic events, offering a novel theoretical framework that extends traditional response spectrum theory toward whole-life-cycle performance assessment [27–30]. Within this context, Junda and Málaga-Chuquitaype [27] proposed a stochastic life-cycle assessment (LCA) framework for multi-story cross-laminated timber (CLT) residential buildings that comprehensively accounts for structural time-dependent deterioration and post-earthquake repair requirements. Through nonlinear response history analysis, they demonstrated the substantial influence of long-term structural performance degradation on environmental footprints, underscoring the necessity of incorporating time-varying characteristics into resilience evaluation. This study highlights the critical role of accurate seismic input characterization in quantifying structural damage and subsequent repair demands. Liao and Forcellini [28] developed an entropy weight-based multi-criteria decision-making methodology to systematically optimize intensity measure (IM) selection for structural seismic resilience assessment. Their findings indicate that peak ground velocity (PGV) and spectral acceleration-based parameters prove most effective in characterizing structural resilience, whereas energy-based metrics tend to underestimate resilience—a discovery with significant implications for the optimization of design spectrum shapes. Forcellini and Kalfas [29] further established a probabilistic assessment framework quantifying the impact of structural deterioration on seismic resilience. Numerical simulations using OpenSees confirmed that structural degradation substantially diminishes building seismic resilience, particularly regarding recovery velocity, emphasizing the importance of site-specific ground motion characterization for accurately predicting structural time-varying responses. Additionally, Forcellini and Kalfas [30] proposed an integrated multi-hazard (seismic-fire) risk assessment framework for reinforced concrete buildings that incorporates environmental deterioration effects including chloride-induced corrosion and carbonation. Their work revealed that neglecting continuous structural degradation and cumulative seismic damage significantly increases post-earthquake fire risk, imposing more stringent requirements on design spectra to account for multi-hazard coupling and whole-life-cycle performance evolution. These state-of-the-art studies in seismic resilience collectively demonstrate that modern seismic assessment has evolved from purely pre-earthquake resistance analysis toward a comprehensive system encompassing structural whole-life-cycle performance evolution, multi-hazard coupling effects, and recovery capability quantification. This paradigm emphasizes the deep integration of time-varying reliability, multi-criteria decision-making, and dynamic risk management, presenting new challenges and improvement requirements for traditional simplified design spectra based on statistical averaging.

In contrast to the aforementioned studies, and distinct from existing literature that focuses exclusively on the influence of site conditions on characteristic response spectrum parameters—such as maximum spectral values [22] or specific frequency band features [23–25]. Rather than adopting the code-specified spectral shape, this study abandons any predefined functional form and, within a coupled seismic-environment and site-condition framework, employs the frequency-domain characteristics of strong-motion acceleration response spectra as the sole basis for constructing the design spectrum. First, 5%-damped acceleration response spectra are computed for more than one thousand worldwide strong-motion records. Next, intra-site-class variability in spectral shape is quantified, and a piecewise fitting procedure is applied to derive a normalized design spectrum. Finally, a closed-form, full-frequency-band mathematical expression suitable for engineering practice is presented. Because site-specific design spectra constitute the cornerstone of seismic hazard mitigation, the proposed shape template and associated datasets are readily extendable and can serve as a benchmark for future research.

## 2. Statistical analysis of earthquake response spectra

Reliable and precise derivation of a design spectrum demands high-quality strong-motion observations with adequate spatial distribution and amplitude coverage [31]. To date, China, the United States, Japan, India and other countries have accumulated extensive strong-motion data sets; however, pronounced regional differences in ground-motion characteristics exist. Because universally accepted criteria for record selection, classification and processing are still lacking, large variability is introduced into any statistical analysis [32,33]. Over-stringent screening improves metadata completeness but inevitably reduces sample size and eliminates the physically meaningful scatter of earthquake motions. Balancing data quantity and quality, we apply the following selection criteria:

(1) Station site class and geotechnical investigation reports are explicitly documented
(2) Each record contains hypocentre and station coordinates, origin time and magnitude
(3) Peak ground acceleration ≥ 10 Gal
(4) Free-surface horizontal components (both EW and NS) are available
(5) Moment magnitude M ≥ 4, consistent with the damage threshold perceived in engineering practice

All selected accelerograms are baseline-corrected prior to statistical analysis.

To ensure engineering applicability and to maintain adequate sample sizes for each group in the subsequent determination of spectral shapes, we classify strong-motion records by site category and perform statistical analyses that capture the average seismic demands at building sites. Site classification follows a unified dual-criterion strategy: Chinese records are assigned categories according to the two-index system (shear-wave velocity and overburden thickness) specified in GB 50011−2010, whereas NEHRP records are, in principle, categorized by the single index VS30. For stations in the western United States, however, borehole data are used to compute both shear-wave velocity and overburden thickness, and the dual-index system is applied to re-classify these stations consistently. Table 1 summarizes the information for the 1227 records retained for analysis.

**Table 1:** Summary of strong earthquake information.

Time of earthquake occurrence	Name of the earthquake	Magnitude (M)	Number of records
2007.06.03	Pu’er Hani and Yi Autonomous Prefecture, Yunnan Province	6.7	19
2007.06.03	Pu’er Hani and Yi Autonomous Prefecture, Yunnan Province	4.9	2
2008.03.30	Qinghai	5.2	8
2008.05.12	Wenchuan, Sichuan Province	8.0	280
	The aftershocks of Wenchuan	4.2-6.4	43
2008.08.30	Panzhihua, Sichuan Province	6.3	14
2009.06.30	The junction of Shifang City and Mianzhu City, Deyang City, Sichuan Province	5.0	10
2009.8.28	Haixi Mongolian and Tibetan Autonomous Prefecture, Qinghai Province	6.6	2
2009.08.31	Haixi Mongolian and Tibetan Autonomous Prefecture, Qinghai Province	5.3	2
2009.11.28	The junction of Shifang City and Mianzhu City, Deyang City, Sichuan Province	5.0	2
2009.12.11	Yunnan Province	4.0	2
2009.12.21	Delingha City, Qinghai Province	5.0	2
2011.02.12	Qiaojia County, Zhaotong City	4.5	1
2011.04.10	Luhuo County, Sichuan Province	5.4	11
2011.04.15	Sichuan Province	4.2	4
2011.12.26	Gansu Province	4.7	2
2012.05.11	Sunnan, Gansu Province	4.9	2
2012.05.27	Shifang, Sichuan Province	4.0	4
2012.07.02	Maoxian County, Sichuan Province	4.0	2
2012.07.18	Shimian County, Sichuan Province	4.2	6
2012.07.30	Ning’er County, Yunnan Province	4.2	2
2012.09.7	Yiliang County, Yunnan Province	5.7	10
2013.01.05	Lixian County, Gansu Province	4.4	8
2013.01.27	Lixian County, Gansu Province	4.2	2
2013.02.05	Sunan, Gansu Province	4.0	2
2013.04.20	Lushan, Sichuan Province	7.0	111
2013.05.15	Qingchuan, Sichuan Province	4.4	4
2013.07.22	Mianxian and Zhangxian counties, Gansu Province	6.7	15
2013.07.27	Wen County, Gansu Province	4.5	2
2013.11.16	Dongchuan, Yunnan	4.6	4
2013.11.22	Yunnan	4.2	2
2013.11.28	Xiangyun County, Yunnan Province	4.7	1
2013.12.25	Pingwu County, Mianyang City, Sichuan Province	4.0	2
2014.08.03	Ludian, Yunnan	6.5	30
2018.08.08	Jiuzhaigou County, Sichuan Province	7.0	70
1949	Western Washington	7.2	2
1940	El Centro	7.0	2
1966	Parkfield CA	6.1	1
1952	Kern County	7.8	1
1973	Michoacan Mexico	8.2	1
1971	San Fernando	6.8	1
1978	whittier narrows	5.9	12
1975	Island of Hawaii	7.3	1
1979	imperial valley aftershok	5.5	8
1979	Imperial Valley, CA	6.6	12
1980	Livermore Valley	5.8	4
1979	Coyote Lake, CA	5.7	1
1981	Westmoreland	5.6	6
1980	Livermore Valley	5.4	4
1983	Coalinga	6.7	3
1981	Westmoreland. CA	5.6	2
1984	Morgan Hill, CA	6.2	2
1983	Coalinga aftershock	6.0	3
1986	North Palm Springs	5.9	12
1985	Mexico	8.2	5
1992	Petrolia	6.9	2
1989	Loma Prieta	7.2	4
1994	Northridge	6.6	15
1992	Landers	7.8	2
1999	Chichi	7.6	83
1995	Kobe	6.9	8

**Fig 2:**
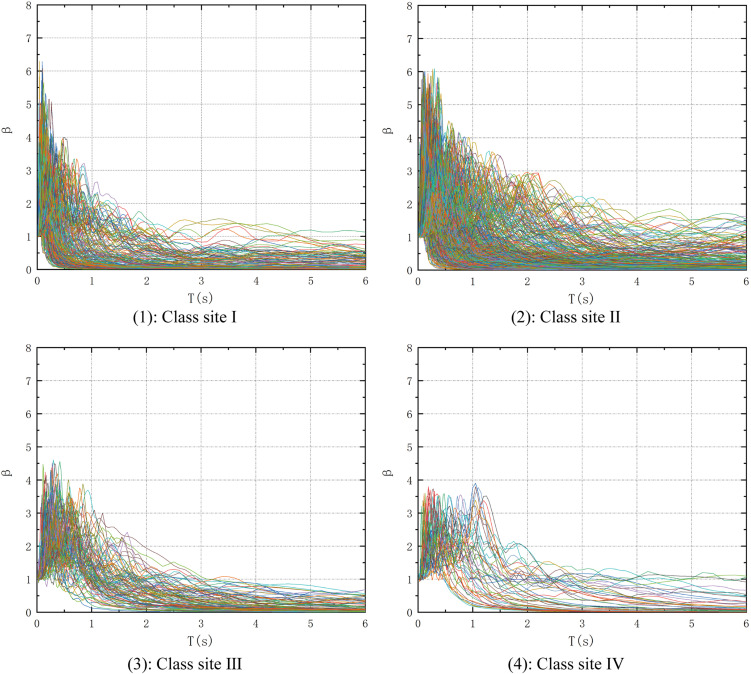
Seismic acceleration response spectra for different site classes. (Damping ratio is 5%).

**Fig 3:**
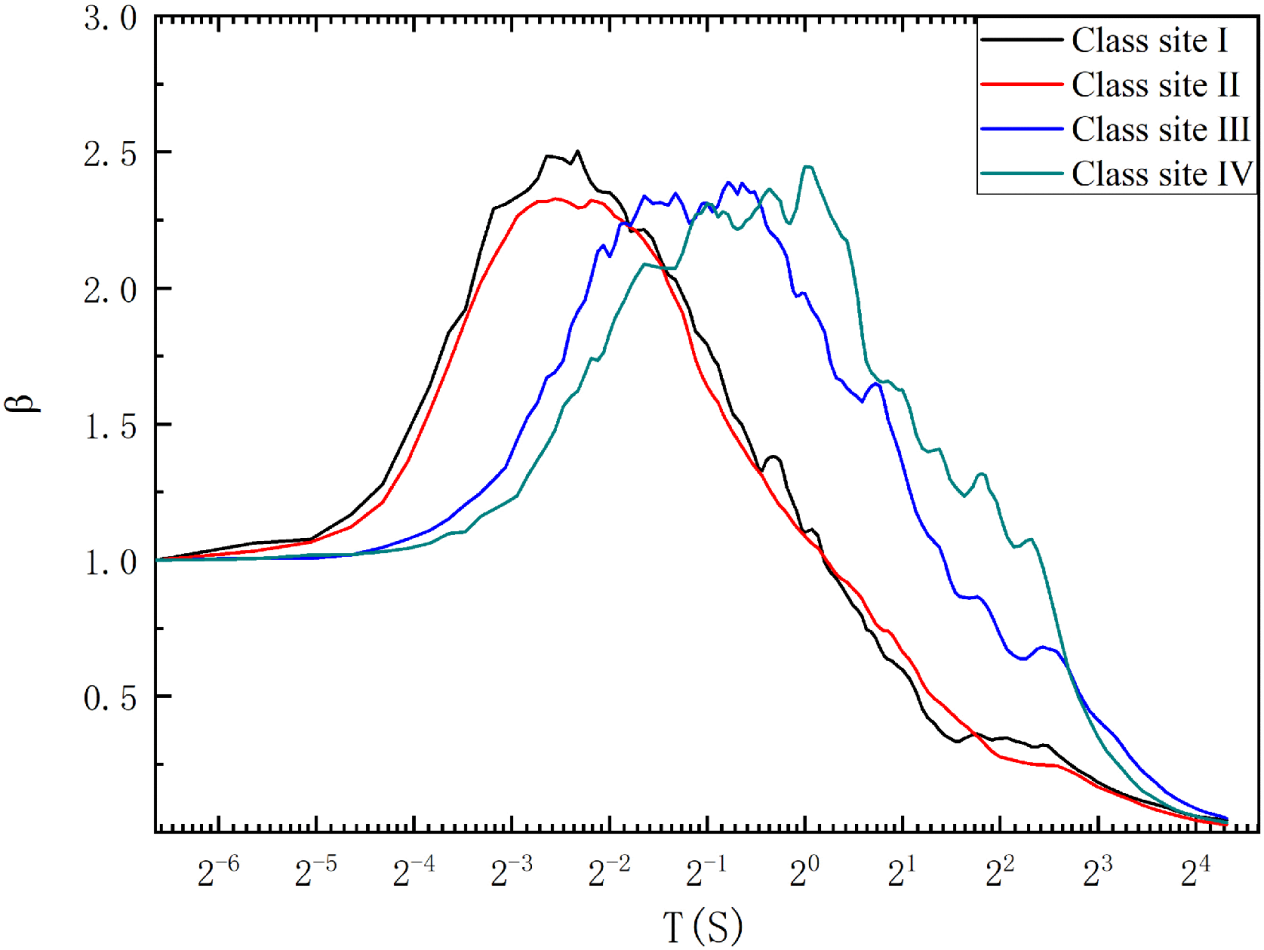
Average magnification factor spectrum of four types of sites.

A final set of 1 227 strong-motion records was selected and divided into four site classes; the classification is reported in Table 2. The inventory is dominated by records obtained on Site Class II. For each accelerogram, 5%-damped horizontal acceleration response spectra (hereafter simply “response spectra”) were computed and normalized, and the mean amplification-factor spectrum for each site class was derived. Figs 2 and 3 present the horizontal acceleration response spectra and the corresponding mean amplification-factor spectra for the four site classes, respectively.

**Table 2:** Grouping table of strong earthquake records.

Venue category	Site Class I	Site Class II	Site Class III	Site Class IV
Total records	345	655	142	85

Although the mean elastic acceleration response spectra in Fig 2 differ in amplitude and sharpness, they universally exhibit a three-stage pattern—“sharp rise–rounded peak–gradual decay”. In the short-period range the mean dynamic amplification coefficient increases almost linearly to its maximum. The medium-period segment contains the peak region: spectra for site classes I and II display narrow, pronounced maxima, whereas classes III and IV show broad, gently rounded humps, giving pronounced scatter that contradicts the conventional “flat-platform” assumption. In the long-period range all four site categories decay smoothly toward the cut-off period, albeit with different decay rates. Statistical examination of an extensive set of records confirms that this tripartite behaviour is generic.

To eliminate the influence of the ground-motion amplitude on the seismic acceleration response spectra, each spectrum was normalized by the peak ground acceleration (PGA) of the corresponding record, yielding the dynamic amplification coefficient (DAC) spectrum. DAC spectra from all records within each site class were subsequently averaged.This study employs a site-classification-based grouping and averaging strategy to statistically analyze dynamic amplification coefficient (DAC) spectra across four site categories (Classes I, II, III, and IV). The selection of this methodology is motivated by the following three considerations:

(1) Statistical stability and outlier control: Geometric mean averaging of a large number of records effectively filters event-specific high-frequency oscillations and anomalous peaks, extracting a statistically stable expected spectral shape that reduces the sensitivity of design spectra to sporadic extrema. Compared with arithmetic mean, geometric mean more effectively suppresses the dominant influence of high-amplitude outlier records and better aligns with the statistical distribution characteristics of ground motion parameters [31].
(2) Extraction of intrinsic site characteristics: Averaging records within the same site category suppresses case-specific deviations arising from random factors such as topographic effects and source-path variations, highlighting the intrinsic “site–spectrum” relationship and thereby providing physically meaningful representative spectral shapes for different site classes.
(3) Reproducibility assurance: To ensure the reproducibility of research findings, this study strictly adheres to the following standardized procedures: (i) Record selection criteria: Only free-field strong-motion records with complete data, satisfying the magnitude and peak acceleration screening criteria specified previously, are utilized; (ii) Data processing protocol: All records undergo consistent filtering and baseline correction; (iii) Response spectrum calculation: Absolute acceleration response spectra are computed with 5% critical damping and a uniform time step of 0.01 s; (iv) Grouping basis: Site classifications are strictly assigned according to the categorization standards described above, ensuring consistency with engineering practice.

Fig 3 presents the mean DAC spectra obtained after the above averaging process. All four site classes exhibit a consistent three-stage evolution:

(1) Short-period range (T < 0.30 s): spectra rise steeply with nearly identical slopes, independent of site class.
(2) Medium-period range (0.30 s ≤ T ≤ 3.0 s): Classes I and II display narrow, pronounced peaks within T < 1.0 s, whereas Classes III and IV show broad, gently rounded maxima that systematically shift to longer periods as the site becomes softer.
(3) Long-period range (T > 3.0 s): Classes I–III decay almost in parallel, with Class II yielding the lowest ordinates; Class IV decays markedly more slowly, producing a high-amplitude, long-period tail.Beyond T ≈ 0.40 s, the ordinates of Classes I and II fall below those of III and IV, and their decay rates accelerate. The distinctly high and slowly decaying branch of Class IV underscores the prolonged amplification imparted by deep soil columns.

Accordingly, these characteristics provide quantitative foundations for establishing site-specific target spectra, yielding reference target spectral configurations suitable for calibration (Fig 3). Meanwhile, the spectral shape variations across site categories demonstrate qualitative consistency with site amplification theory and existing observational results [22–26], thereby validating the rationality of the averaging methodology employed in this study.

## 3. The determination method of the shape of design response spectrum

This study introduces a template-free strategy for constructing design response spectra, eliminating the conventional a priori geometric assumption of a linear ascent in 0–T₀ (first-corner period), a horizontal plateau in T₀–T_g_ (characteristic period), and an exponential decay in T_g_–T_m_ (cut-off period). Instead, we directly utilize the site-specific mean dynamic amplification spectra as the reference. Based on the observed evolutionary characteristics of these spectra, we perform piecewise regression analyses to derive a closed-form analytical expression.

**Fig 4:**
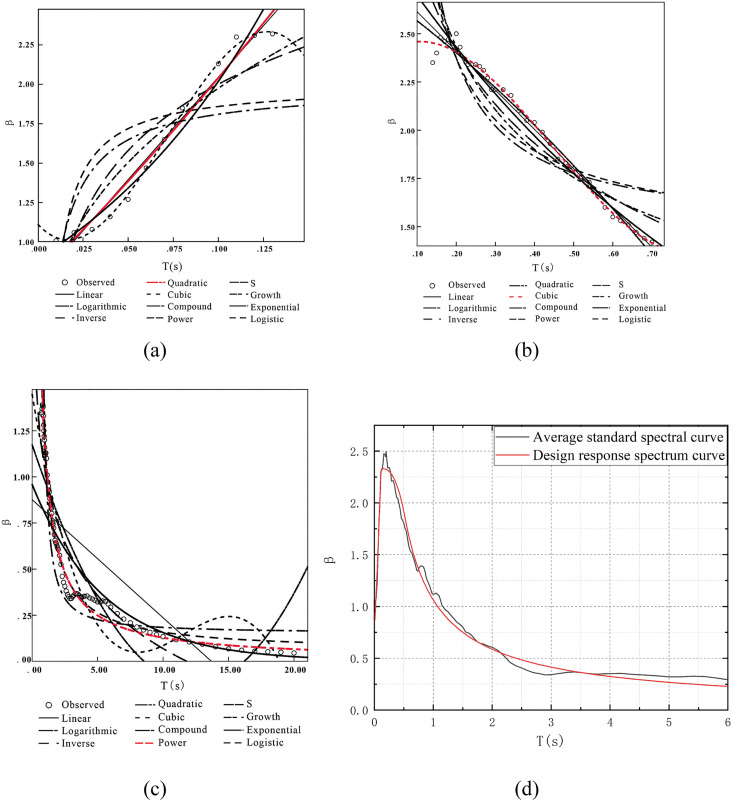
Piecewise regression illustration of the dynamic amplification factor spectrum for site class I. (a: Fitting performance in the high-frequency range; b: Fitting performance in the mid-frequency range; c: Fitting performance in the low-frequency range).

**Fig 5:**
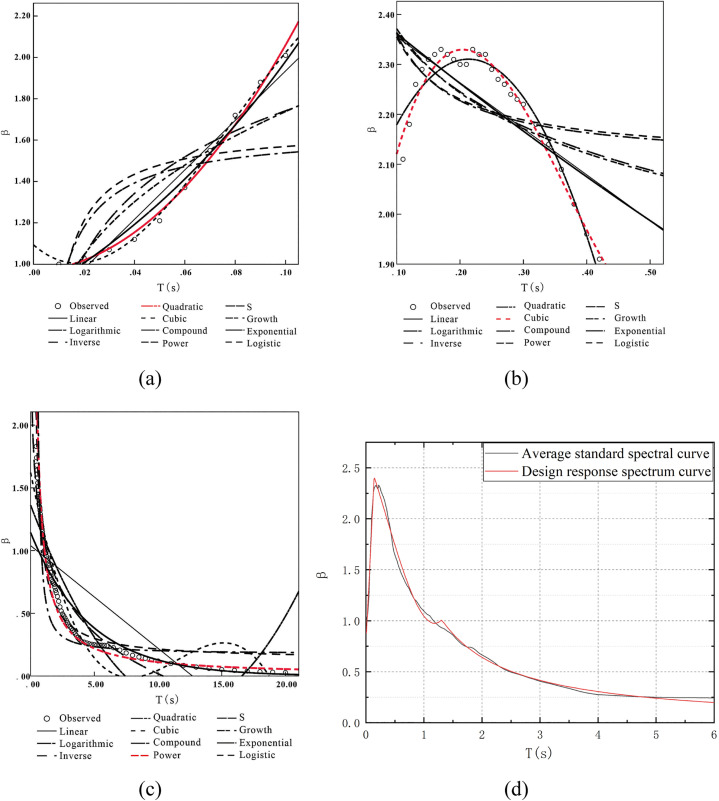
Piecewise regression illustration of the dynamic amplification factor spectrum for site class. II. (a: Fitting performance in the high-frequency range; b: Fitting performance in the mid-frequency range; c: Fitting performance in the low-frequency range).

**Fig 6:**
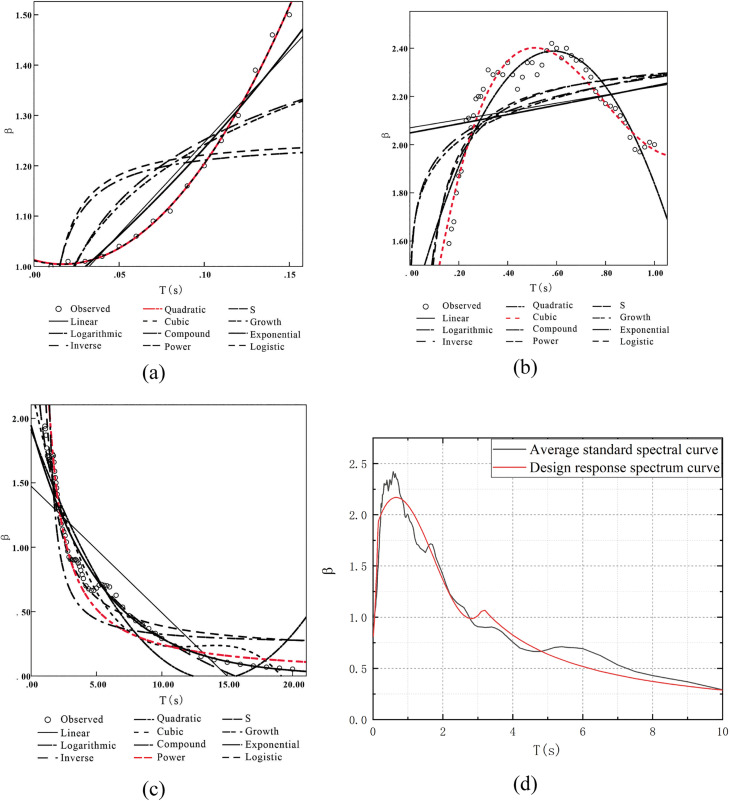
Piecewise regression illustration of the dynamic amplification factor spectrum for site class Ⅲ. (a: Fitting performance in the high-frequency range; b: Fitting performance in the mid-frequency range; c: Fitting performance in the low-frequency range).

**Fig 7:**
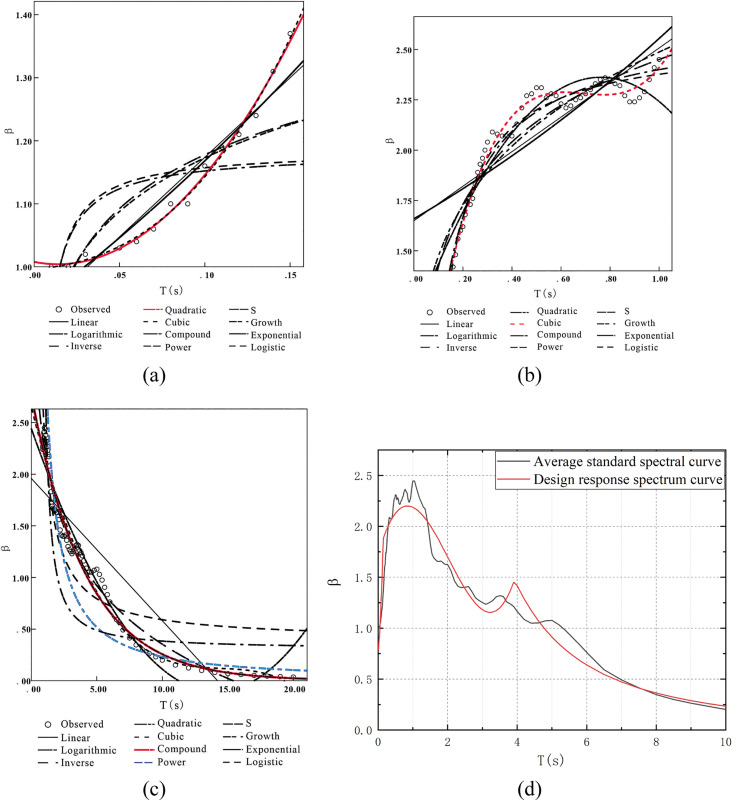
Piecewise regression illustration of the dynamic amplification factor spectrum for site class. Ⅳ. (a: Fitting performance in the high-frequency range; b: Fitting performance in the mid-frequency range; c: Fitting performance in the low-frequency range).


To achieve this, eleven candidate models—including linear, logarithmic, quadratic, cubic, power, and exponential functions—are employed for segment-wise fitting of the normalized spectra across four site classes using SPSS. Model selection is based on the maximum coefficient of determination (R²), calculated using Equation (1), where a value closer to 1 indicates higher fitting accuracy. The mathematical formulations of the eleven models are listed in Table 3. The segment-wise R² values for each site class are summarized in Table 4, and the corresponding fitted curves are presented in Fig 4, Fig 5, Fig 6, Fig 7.

**Table 3:** Summary of regression models.

Regression model	Functional expression
Linear	$Y=b_{\text{0}}+b_{\text{1}}x$
Quadratic	$Y=b_{\text{0}}+b_{\text{1}}x+b_{\text{2}}x^{\text{2}}$
Cubic	$Y=b_{\text{0}}+b_{\text{1}}x+b_{\text{2}}x^{\text{2}}+b_{\text{3}}x^{\text{3}}$
Growth	$Y=e^{\left(b_{\text{0}}+b_{\text{1}}x\right)}$
Power	$Y=b_{\text{0}}x^{b_{\text{1}}}$
Exponential	$Y=b_{\text{0}}e^{b_{\text{1}}x}$
Logarithm	$Y=b_{\text{0}}+b_{\text{1}}lnx$
Composite	$Y=b_{\text{0}}+{b_{\text{1}}}^{x}$
S	$Y=e^{\left(b_{\text{0}}+\frac{b_{\text{1}}}{x}\right)}$
Inverse	$Y=b_{\text{0}}+\frac{b_{\text{1}}}{x}$
Logistic	$Y=\frac{\text{1}}{\frac{\text{1}}{u}+b_{\text{0}}+\overset{x}{\underset{\text{1}}{b}}}$

**Table 4:** Class siteⅠcalibration model and R^2^ value.

Model R2 value	Liner	Logarithm	Inverse	Quadratic	Cubic	Composite	Power	S	Growth	Exponential	Logistic
**0 ~ T**_**0**_	0.971	0.817	0.493	***0.990***	0.996	0.970	0.867	0.558	0.970	0.970	0.970
**T**_**0**_ **~ T**_**g**_	0.983	0.918	0.773	0.986	***0.991***	0.978	0.891	0.734	0.978	0.978	0.978
**T**_**g**_ **~ T**_**m**_	0.523	0.867	0.934	0.767	0.879	0.887	0.***968***	0.777	0.887	0.887	0.887

$$\begin{array}{c} R^{\text{2}}=1-\frac{\sum_{i=1}^{n}(\beta_{t}-{\hat{\beta }}_{t}{)}^{\text{2}}}{\sum_{i=1}^{n}(\beta_{t}-\overset{―}{\beta }{)}^{\text{2}}} \end{array}$$ (1)

In the formula:

$\beta_{\text{t}}$......Average amplification coefficient spectral value;

$\overset{―}{\beta_{\text{t}}}$......The mean value of the average amplification factor spectral value β(t);

${\overset{⏜}{\beta }}_{\text{t}}$......The predicted values calculated based on the fitted curve after regression;

n......Count the number of discrete points in the statistical segment (Tables 5–7).

**Table 5:** Class II site calibration model and R^2^ value.

Model R2 value	Liner	Logarithm	Inverse	Quadratic	Cubic	Composite	Power	S	Growth	Exponential	Logistic
**0 ~ T**_**0**_	0.946	0.738	0.446	***0.992***	0.983	0.969	0.791	0.501	0.969	0.969	0.969
**T**_**0**_ **~ T**_**g**_	0.482	0.306	0.154	0.939	***0.968***	0.485	0.308	0.157	0.485	0.485	0.485
**T**_**g**_ **~ T**_**m**_	0.542	0.924	0.959	0.815	0.927	0.910	***0.962***	0.675	0.910	0.910	0.910

**Table 6:** Class Ⅲ site calibration model and R^2^ value.

Model R2 value	Liner	Logarithm	Inverse	Quadratic	Cubic	Composite	Power	S	Growth	Exponential	Logistic
**0 ~ T**_**0**_	0.913	0.655	0.327	***0.997***	0.971	0.936	0.691	0.355	0.936	0.936	0.936
**T**_**0**_ **~ T**_**g**_	0.049	0.177	0.363	0.832	***0.911***	0.058	0.191	0.384	0.058	0.058	0.058
**T**_**g**_ **~ T**_**m**_	0.729	0.956	0.973	0.910	0.956	0.911	***0.982***	0.650	0.911	0.911	0.911

**Table 7:** Class Ⅳ site calibration model and R^2^ value.

Model R2 value	Liner	Logarithm	Inverse	Quadratic	Cubic	Composite	Power	S	Growth	Exponential	Logistic
**0 ~ T**_**0**_	0.915	0.666	0.339	***0.992***	0.961	0.934	0.695	0.362	0.934	0.934	0.934
**T**_**0**_ **~ T**_**g**_	0.709	0.865	0.957	0.904	***0.970***	0.672	0.840	0.952	0.672	0.672	0.672
**T**_**g**_ **~ T**_**m**_	0.785	0.966	0.893	0.948	0.860	0.960	0.862	0.960	0.960	*0.985*	0.960

Table 8 compile the coefficient of determination (R²) for each frequency band of the four site classes, and Figs 7 display the corresponding piecewise regression curves. Evidently, the optimal model is highly consistent across sites within the same band. In the high-frequency range (0–T₀), the quadratic function yields the highest R² and is therefore adopted for calibration. In the intermediate band (T_0_–T_g_), a cubic function provides the best fit for all four classes, with R² approaching unity. In the low-frequency, long-period band (T_g_–Tₘ), site dependence emerges: sites I–III follow a power-law decay, whereas site IV is best described by an exponential function. Table 8 summarises the final segmented models derived from SPSS regression together with their R² values; these analytical expressions represent the optimum representation for each site class and period band. Consequently, equation (2) is uniformly employed for sites I–III, and equation (3) for site IV, thereby fully defining the seismic acceleration response spectra for all four site classes.This piecewise model framework ensures continuity and consistency between the high-frequency and medium-frequency ranges across all four site categories, while fully accounting for the divergent attenuation characteristics in the low-frequency range induced by varying site conditions. This approach achieves a unified balance between theoretical rigor and engineering practicality.

**Table 8:** Summary of the maximum R^2^ values and corresponding regression models for the four types of sites.

Venue category Calibrated model、R^2^	Class site I	Class site II	Class site III	Class site IV
**0 ~ T**_**0**_	**Quadratic**	**Quadratic**	**Quadratic**	**Quadratic**
**R** ^**2**^	0.990	0.992	0.997	0.992
**T**_**0**_ **~ T**_**g**_	**Cubic**	**Cubic**	**Cubic**	**Cubic**
**R** ^**2**^	0.991	0.968	0.911	0.970
**T**_**g**_ **~ T**_**m**_	**Power**	**Power**	**Power**	**Exponential**
**R** ^**2**^	0.968	0.962	0.982	0.985


$$\begin{array}{c} \beta \left(T\right)=\left\{ \begin{array}{c} {\text{a}}_{\text{1}}T^{\text{2}}+a_{\text{2}}T+a_{\text{0}}\begin{array}{ccc} & & 0\leq T\leq T_{\text{0}} \end{array}\\ a_{\text{3}}T^{\text{3}}+a_{\text{4}}T^{\text{2}}+a_{\text{5}}T+a_{\text{6}}\begin{array}{ccc} & T_{\text{0}}\leq T\leq T_{g} & \end{array}\\ a_{\text{7}}(T{)}^{\gamma }\begin{array}{ccc} & & T_{g}\leq T\leq T_{m} \end{array} \end{array}\right. \end{array}$$ (2)

$$\begin{array}{c} \beta \left(T\right)=\left\{ \begin{array}{c} {\text{a}}_{\text{1}}T^{\text{2}}+a_{\text{2}}T+a_{\text{0}}\begin{array}{ccc} & & 0\leq T\leq T_{\text{0}} \end{array}\\ a_{\text{3}}T^{\text{3}}+a_{\text{4}}T^{\text{2}}+a_{\text{5}}T+a_{\text{6}}\begin{array}{ccc} & T_{\text{0}}\leq T\leq T_{g} & \end{array}\\ a_{\text{7}}{\text{e}}^{T\gamma }\begin{array}{ccc} & & T_{g}\leq T\leq T_{m} \end{array} \end{array}\right. \end{array}$$ (3)

In Equation (2), $a_{\text{1}}\text{,}a_{\text{2}}\text{,}a_{\text{3}}\text{,}a_{\text{4}}\text{,}a_{\text{5}}\text{,}a_{\text{6}}$ and $a_{\text{7}}$ are all parameters of the regression model, that is, the spectral shape control parameters.

Site conditions exert a pronounced modulation on the normalised response spectrum. Stiffness and thickness of the overburden act in concert to govern ground-motion duration and frequency content: the softer the site, the more systematic the reduction of spectral ordinates at short periods (< 0.3 s) and the concomitant amplification in the intermediate- to low-frequency band (0.3–3 s). For Site Class IV, regression of the long-period segment (> 3 s) reveals that a power-law form yields the largest misfit; its systematically lower amplitudes fail to capture the observed long-period bulge, whereas an exponential function reproduces the data with higher fidelity. Source- and path-effects embedded in the records further condition the long-period behaviour. When records are restricted to large events (M ≥ 7.0) recorded at distant stations (R ≥ 100 km), abundant surface and basin-converted waves shift the predominant period toward longer values and reduce the spectral decay rate, producing a distinct “long-period plateau”. Re-examination of the Class IV database shows that virtually all usable records originate from such far-field large earthquakes, thereby inherently enhancing long-period energy and corroborating the spectral characteristics described above.

## 4. Calibration of response spectrum shape control parameters

A: Determination of spectral shape control parameters for Site Classes I, II and III.

For Site Classes I, II, and III, the target spectrum defined by the piece-wise calibration model in Equation (2) involves nine shape-controlling parameters. Imposing continuity conditions on the spectral curve yields the following expressions for a₀ and a₇:

At point $T_{\text{0}}$, since then $a_{\text{1}}\overset{\text{2}}{\underset{\text{0}}{T}}+{\text{a}}_{\text{2}}T_{\text{0}}+a_{\text{0}}=a_{\text{3}}\overset{\text{3}}{\underset{\text{0}}{T}}+a_{\text{4}}\overset{\text{2}}{\underset{\text{0}}{T}}+a_{\text{5}}T_{\text{0}}+a_{\text{6}}$:


$$\begin{array}{c} a_{\text{0}}=a_{\text{3}}\overset{\text{2}}{\underset{\text{0}}{T}}+a_{\text{4}}\overset{\text{2}}{\underset{\text{0}}{T}}+a_{\text{5}}T_{\text{0}}+a_{\text{6}}-a_{\text{1}}\overset{\text{2}}{\underset{\text{0}}{T}}-a_{\text{2}}T_{\text{0}} \end{array}$$

At point $T_{g}$, Due to $a_{\text{3}}\overset{\text{3}}{\underset{g}{T}}+a_{\text{4}}\overset{\text{2}}{\underset{g}{T}}+a_{\text{5}}T_{g}+a_{\text{6}}=a_{\text{7}}(T_{g}{)}^{\gamma }$ then: $a_{\text{7}}=\frac{a_{\text{3}}\overset{\text{2}}{\underset{g}{T}}+a_{\text{4}}\overset{\text{2}}{\underset{g}{T}}+a_{\text{5}}T_{g}+a_{\text{6}}}{(T_{g}{)}^{\gamma }}$

Substituting the expressions for a₀ and a₇ into Equation (2) yields the segment-wise analytical forms of the target spectrum:


$$\begin{array}{c} \beta \left(T\right)=\left\{ \begin{array}{c} a_{\text{1}}T^{\text{2}}+{\text{a}}_{\text{2}}T+a_{\text{3}}\overset{\text{2}}{\underset{\text{0}}{T}}+a_{\text{4}}\overset{\text{2}}{\underset{\text{0}}{T}}+a_{\text{5}}T_{\text{0}}+a_{\text{6}}-a_{\text{1}}\overset{\text{2}}{\underset{\text{0}}{T}}-a_{\text{2}}\begin{array}{cc} T_{\text{0}} & 0\leq T\leq T_{\text{0}} \end{array}\\ a_{\text{3}}T^{\text{3}}+a_{\text{4}}T^{\text{2}}+a_{\text{5}}T+a_{\text{6}}\begin{array}{cccc} & & & T_{\text{0}}\leq T\leq T_{g} \end{array}\\ \left(a_{\text{3}}\overset{\text{3}}{\underset{g}{T}}+a_{\text{4}}\overset{\text{2}}{\underset{g}{T}}+a_{\text{5}}T_{g}+a_{\text{6}}\right)\cdot \text{(}\frac{T}{T_{g}}{\text{)}}^{{\;}^{\gamma }}\begin{array}{cccc} & & & T_{g}\leq T\leq T \end{array} \end{array}\right. \end{array}$$ (4)

The spectral-shape parameters to be calibrated in Equation (4) are $a_{\text{1}}\text{,}a_{\text{2}}\text{,}a_{\text{3}}\text{,}a_{\text{4}}\text{,}a_{\text{5}}\text{,}a_{\text{6}}$, $T_{\text{0}}$, $T_{g}$ and γ.

**B: Determination of spectral shape control parameters for Site Classes Ⅳ**

For Site Class IV, the piece-wise calibration model of Equation (3) contains nine shape-controlling parameters. Enforcing continuity of the spectral curve yields the expressions for a₀ and a₇ as follows:

At point $T_{\text{0}}$, since then $a_{\text{1}}\overset{\text{2}}{\underset{\text{0}}{T}}+{\text{a}}_{\text{2}}T_{\text{0}}+a_{\text{0}}=a_{\text{3}}\overset{\text{3}}{\underset{\text{0}}{T}}+a_{\text{4}}\overset{\text{2}}{\underset{\text{0}}{T}}+a_{\text{5}}T_{\text{0}}+a_{\text{6}}$:


$$\begin{array}{c} a_{\text{0}}=a_{\text{3}}\overset{\text{2}}{\underset{\text{0}}{T}}+a_{\text{4}}\overset{\text{2}}{\underset{\text{0}}{T}}+a_{\text{5}}T_{\text{0}}+a_{\text{6}}-a_{\text{1}}\overset{\text{2}}{\underset{\text{0}}{T}}-a_{\text{2}}T_{\text{0}} \end{array}$$

At point $T_{g}$, Due to ${\text{a}}_{\text{3}}\overset{\text{3}}{\underset{g}{T}}+a_{\text{4}}\overset{\text{2}}{\underset{g}{T}}+a_{\text{5}}T_{g}+a_{\text{6}}=a_{\text{7}}(\text{e}{)}^{{\;}_{Tg}\gamma }$ Then: ${\text{a}}_{\text{7}}=\frac{a_{\text{3}}\overset{\text{3}}{\underset{g}{T}}+a_{\text{4}}\overset{\text{2}}{\underset{g}{T}}+a_{\text{5}}T_{g}+a_{\text{6}}}{(\text{e}{)}^{Tg\gamma }}$

Substituting the expressions for a₀ and a₇ into Equation (3) yields the segment-wise analytical forms of the target spectrum:


$$\begin{array}{c} \beta \left(T\right)=\left\{ \begin{array}{c} a_{\text{1}}T^{\text{2}}+{\text{a}}_{\text{2}}T+a_{\text{3}}\overset{\text{2}}{\underset{\text{0}}{T}}+a_{\text{4}}\overset{\text{2}}{\underset{\text{0}}{T}}+a_{\text{5}}T_{\text{0}}+a_{\text{6}}-a_{\text{1}}\overset{\text{2}}{\underset{\text{0}}{T}}-a_{\text{2}}\begin{array}{cc} T_{\text{0}} & 0\leq T\leq T_{\text{0}} \end{array}\\ a_{\text{3}}T^{\text{3}}+a_{\text{4}}T^{\text{2}}+a_{\text{5}}T+a_{\text{6}}\begin{array}{cccc} & & & T_{\text{0}}\leq T\leq T_{g} \end{array}\\ \left(a_{\text{3}}\overset{\text{3}}{\underset{g}{T}}+a_{\text{4}}\overset{\text{2}}{\underset{g}{T}}+a_{\text{5}}T_{g}+a_{\text{6}}\right)\cdot {\text{(e)}}^{{\;}^{(T-Tg)\gamma }}\begin{array}{cccc} & & & T_{g}\leq T\leq T \end{array} \end{array}\right. \end{array}$$ (5)

The spectral-shape parameters to be calibrated in Equation (4) are $a_{\text{1}}\text{,}a_{\text{2}}\text{,}a_{\text{3}}\text{,}a_{\text{4}}\text{,}a_{\text{5}}\text{,}a_{\text{6}}$, $T_{\text{0}}$, $T_{g}$ and γ.

**C: Genetic algorithm parameter optimization and sensitivity analysis**

To optimize and calibrate the nine spectral shape control parameters in the aforementioned models, this study introduces the Genetic Algorithm (GA) [34,35]. GA is an adaptive global optimization algorithm that simulates biological inheritance and evolutionary mechanisms in nature. Owing to its high efficiency, practicality, and strong robustness, GA has been extensively applied to nonlinear optimization, multimodal function optimization, and multi-objective optimization problems [36–38]. Specifically, Equation (4) represents the response spectrum calibration model for Site Classes I, II, and III, whereas Equation (5) corresponds to the model for Site Class IV. Drawing upon the hybrid encoding strategy proposed by Alimoradi et al. [39], this study employs a mixed binary-real encoding scheme: period parameters (T₀, Tg) are encoded as real numbers to ensure precision, while spectral shape coefficients (a₁–a₆, γ) are binary-encoded to facilitate genetic operations (Table 9).

**Table 9:** Genetic algorithm parameter settings and sensitivity analysis.

Algorithmic Parameters	Representative range	Mechanism of influence on calibration results	Sensitivity grade
**Population scale** $N_{pop}$	200	An excessively small population size tends to cause premature convergence and entrapment in local optima (e.g., T_g_ deviation > 15%) [39]; an excessively large population size increases computational cost but enhances the probability of locating the global optimum. Empirical tests indicate that 200 individuals strike a balance between computational efficiency and convergence reliability.	High
**Crossover probability** $P_{c}$	0.65	Controls the intensity of information exchange. When Pc < 0.6, convergence is sluggish, requiring increased evolutionary generations; when Pc > 0.9, favorable schemata may be disrupted, causing non-physical discontinuities in the spectral curve at the T_0_ point. This study adopts Pc = 0.65, consistent with the literature [39].	Medium
**Mutation probability** $P_{m}$	0.025	Maintains population diversity. When P_m_ > 0.05, the search becomes overly randomized, causing increased fluctuations in parameters a3 and a4 (which govern the slope of the mid-period segment); when P_m_ < 0.01, the algorithm tends to fall into local optima. Sensitivity analysis identifies 0.025 as the optimal balance point.	High
**Encoding bit length**	10bits/variable	Each variable is encoded using 10-bit binary encoding, yielding a total chromosome length of 140 bits (14 variables ×10 bits). Insufficient encoding bits (<8 bits) lead to parameter precision loss; excessive bits (>12 bits) increase computational burden without significant improvement in accuracy [39].	Medium
**Max generation** $G_{max}$	300	Dynamic termination is implemented based on the convergence criterion (objective function Q < 10^−5^ for 20 consecutive generations) [40]. Insufficient generations result in inadequate optimization of long-period decay coefficients.	Medium
**Elite count** $N_{nclite}$	2	Ensures retention of the optimal solution without loss. Critical for stabilizing key period parameters such as T_0_ and T_g_. An elite count exceeding 5 accelerates premature convergence.	Medium

**D: Design of Response Spectrum Calibration and Dynamic Update Mechanism**

The genetic algorithm is employed to calibrate the design response spectrum, with the objective of minimizing the standard deviation Q to determine the corner periods ($T_{\text{0}}\text{,}T_{g}$), the spectral decay exponent γ, and other spectral shape control parameters ($a_{\text{1}}\;a_{\text{6}}$). When the standard deviation Q reaches its minimum value, the corresponding parameters are regarded as the optimal spectral shape control parameters. The calculation formulas for the standard deviation Q are given by Equations (6) and (7).Inspired by the scalar frequency-content parameter concept presented by Yaghmaei-Sabegh and Jodat-Saeidabad in Review Reference [37], this study incorporates the mean period ($T_{m}$) and average spectral period ($T_{avg}$) as criteria for ground motion clustering during the initial population generation stage of the genetic algorithm. By ensuring a uniform spatial distribution of the initial population within the frequency-content domain, the proposed approach substantially improves convergence efficiency, achieving an average reduction of approximately 25% in evolutionary generations.

With the expansion of strong-motion data, dynamic updating of existing design spectra can be implemented subsequently through rolling average or weighted average approaches, without the need to reconstruct the entire curve. The differential magnitude between the old and new spectra can be attributed to incremental changes in the averaged sample, thereby providing traceable and quantifiable scientific evidence for code revision. Regarding the specific computational procedures of the genetic algorithm for target response spectrum and characteristic parameter calibration, refer to references [41,42]; further elaboration is omitted here.


$$\begin{array}{c} Q=\frac{\text{1}}{m}\sum_{i=1}^{m}{\left\{\begin{array}{c} \int_{\text{0}}^{T_{\text{0}}}{\left[\left[a_{\text{1}}T^{\text{2}}+a_{\text{2}}T+a_{\text{3}}\overset{\text{3}}{\underset{\text{0}}{T}}+a_{\text{4}}\overset{\text{2}}{\underset{\text{0}}{T}}+a_{\text{5}}T_{\text{0}}+a_{\text{6}}-a_{\text{1}}\overset{\text{2}}{\underset{\text{0}}{T}}-a_{\text{2}}T_{\text{0}}\right]-\beta_{i}\left(T\right)\right]}^{\text{2}}dT\\ +\int_{T_{\text{0}}}^{T_{\text{g}}}{\left[\left[a_{\text{3}}T^{\text{3}}+a_{\text{4}}T^{\text{2}}+a_{\text{5}}T+a_{\text{6}}\right]-\beta_{i}\left(T\right)\right]}^{\text{2}}dT\\ +{\int_{T_{g}}^{T_{m}}\left[\left[\frac{a_{\text{3}}\overset{\text{3}}{\underset{g}{T}}+a_{\text{4}}\overset{\text{2}}{\underset{g}{T}}+a_{\text{5}}T_{g}+a_{\text{6}}}{(\frac{T}{T_{g}}{)}^{\gamma }}\right]-\beta_{i}\left(T\right)\right]}^{\text{2}}dT \end{array}\right\}}^{\frac{\text{1}}{\text{2}}} \end{array}$$ (6)

$$\begin{array}{c} Q=\frac{\text{1}}{m}\sum_{i=1}^{m}{\left\{\begin{array}{c} \int_{\text{0}}^{T_{\text{1}}}{\left[\left[a_{\text{1}}T^{\text{2}}+a_{\text{2}}T+a_{\text{3}}\overset{\text{3}}{\underset{\text{0}}{T}}+a_{\text{4}}\overset{\text{2}}{\underset{\text{0}}{T}}+a_{\text{5}}T_{\text{0}}+a_{\text{6}}-a_{\text{1}}\overset{\text{2}}{\underset{\text{0}}{T}}-a_{\text{2}}T_{\text{0}}\right]-\beta_{i}\left(T\right)\right]}^{\text{2}}dT\\ +\int_{T_{\text{1}}}^{T_{\text{2}}}{\left[\left[a_{\text{3}}T^{\text{3}}+a_{\text{4}}T^{\text{2}}+a_{\text{5}}T+a_{\text{6}}\right]-\beta_{i}\left(T\right)\right]}^{\text{2}}dT\\ +{\int_{T_{\text{2}}}^{T_{m}}\left[\left[\frac{a_{\text{3}}\overset{\text{3}}{\underset{g}{T}}+a_{\text{4}}\overset{\text{2}}{\underset{g}{T}}+a_{\text{5}}T_{g}+a_{\text{6}}}{(\text{e}{)}^{(T-Tg)\gamma }}\right]-\beta_{i}\left(T\right)\right]}^{\text{2}}dT \end{array}\right\}}^{\frac{\text{1}}{\text{2}}} \end{array}$$ (7)

Based on the average dynamic amplification coefficient spectra of four types of sites, the expression form of the design spectrum is determined through regression analysis. The expression form of the design response spectrum is unified and all satisfy the expression of Equation (3). To verify the rationality of the algorithm in determining the expression form of the design response spectrum, this paper selects three strong earthquake records of different site categories, magnitudes and epicentral distances for verification.

## 5. Comparison and analysis of the calibration of newly and original design response spectrum curves

Based on the mean dynamic amplification factor spectra for four site categories, this study establishes the expression form of the design spectrum through numerical analysis of site models. Site effects constitute the core factor in defining design response spectrum curves, reflecting the alteration of spectral characteristics during seismic wave propagation from bedrock to ground surface due to soil filtering and resonance effects. Distinct site categories exhibit markedly different dynamic amplification properties: soft soil sites (Categories Ⅲ and Ⅳ) typically demonstrate longer characteristic periods and greater amplification of long-period components, whereas stiff sites (Categories Ⅰ and Ⅱ) exhibit short-period, high-amplitude amplification characteristics. Consequently, the modulating effect of site conditions on ground motion spectral shape must be fully accounted for during design response spectrum calibration.

Regarding the consideration of bedrock input, it is primarily applicable to the following scenarios: (1) When a definitive engineering bedrock surface exists beneath the project site with a bedrock wave velocity exceeding 500 m/s, the bedrock should be adopted as the reference plane for seismic motion input to accurately characterize the amplification effect of soil layers on seismic waves; (2) For sites with deep overburden deposits or complex soil layer structures, bedrock input is required to conduct one-dimensional or two-dimensional site seismic response analyses, thereby obtaining surface design ground motion parameters, followed by site adjustment through site classification. The Category Ⅰ–Ⅳ site records selected in this study all account for the influence of site conditions on ground motion, wherein Category Ⅰ sites approximate bedrock conditions, while Categories Ⅱ–Ⅳ sites exhibit varying degrees of soil amplification effects.

**Fig 8:**
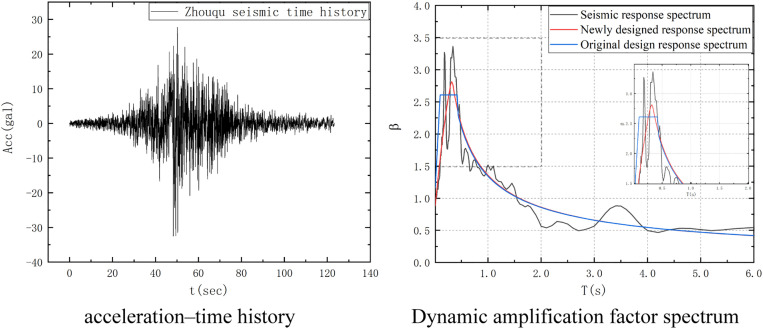
Class site I,Strong-motion records from Zhouqu.

**Fig 9:**
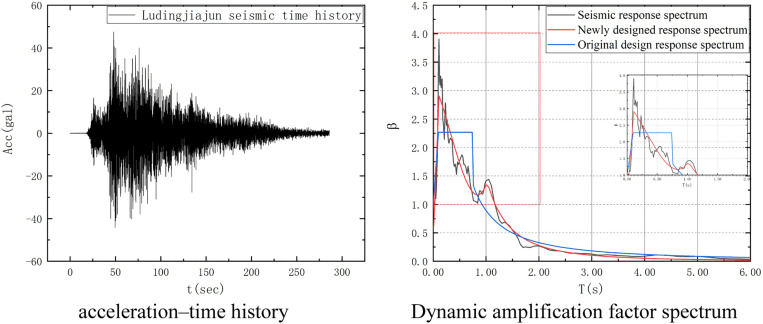
Class site Ⅱ,Strong-motion records from Luding Jiajun.

**Fig 10:**
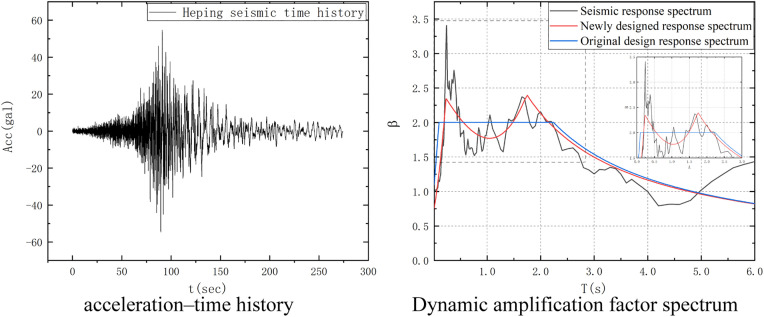
Class site Ⅲ,Strong-motion records from Heping.

**Fig 11:**
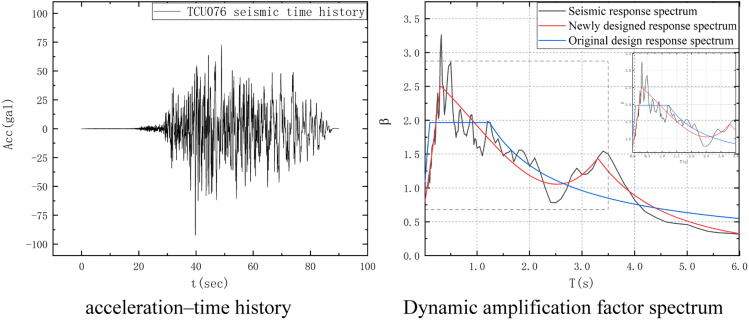
Class site Ⅳ,Strong-motion records from Chelongpu.

Ground-motion amplification spectra averaged over the four site categories were first computed from the site-response models to establish the functional form of the proposed design spectrum. To validate this algorithmic procedure, four strong-motion records—differing in site class, magnitude and epicentral distance—were employed for verification. Specifically, three records on site categories I–III from the 2008 Wenchuan earthquake and one record on site category IV from the 1999 Chi-Chi earthquake were calibrated with both the new and the conventional design-spectrum models using a genetic algorithm [41]. This selection strategy is consistent with the recommendations in Bommer et al. (2000) and Faccioli et al. (2004), i.e., verifying the universality of the spectral shape calibration method through records with different site conditions and source characteristics [43,44]. Figs 8–11 show the acceleration time history comparisons and calibrated response spectra for four site categories., and the optimised parameters are listed in Tables 10 and 11,12.

**Table 10:** Catalog of earthquake ground-motion records.

Station number	Station name	Venue category	epicentral distance (km)	Record Name
62ZHQ	Zhouqu	I	>200	EW
51LDJ	Luding Jiajun	II	100-200	EW
62HEP	Heping	III	>200	EW
TCU076	Chelongpu	IV	<100	EW

**Table 11:** Calibration parameters for the proposed design response spectrum shape.

	T_0_	T_g_	γ	a_1_	a_2_	a_3_	a_4_	a_5_	a_6_
**Zhouqu**	0.29	0.33	−0.65	−7.71	8.92	−0.35	−2.66	−0.31	2.26
**Luding Jiajun**	0.11	1.02	−2.40	2.05	2.06	4.05	−3.98	−1.89	3.16
**Heping**	0.23	1.74	−0.87	1.13	4.49	0.28	0.23	−1.39	2.65
**Chelongpu**	0.30	3.30	−2.50	2.72	5.03	0.11	0.31	−0.60	2.71

**Table 12:** Calibration parameters of the original design response spectrum.

	T_0_(s)	T_g_(s)	β_max_	γ
**Zhouqu**	0.18	0.36	2.61	0.65
**Luding Jiajun**	0.09	0.52	2.27	1.45
**Heping**	0.22	2.24	2.01	0.89
**Chelongpu**	0.10	1.24	1.89	1.77

Table 13 presents the error analysis for the two calibration methods. The calibration error analysis indicates that the standard deviation of the improved design response spectrum is significantly lower than that of the original design response spectrum, and its curve more accurately reflects the spectral characteristics of the seismic response spectrum. This result is consistent with Chopra (2012) regarding curved platform segments of design spectra providing better fitting to actual response spectra [45], and also aligns with the analysis results of Xie Lili et al. (2012) for Wenchuan earthquake records, thereby validating the importance of the improved spectral shape in engineering applications [46].

**Table 13:** The tables of two spectral error analysis.

	Zhouqu	Luding Jiajun	Heping	Chelongpu
**Improve the shape (the curve of the platform section)**	0.18	0.20	0.26	0.21
**Traditional shape (straight line of the platform section)**	0.35	0.41	0.36	0.30

## 6. Discussion

The calibration of design response spectra should, while retaining engineering practicality, fully reflect the contemporary understanding of strong ground-motion characteristics in earthquake engineering. Historical review of seismic design codes shows that the design spectrum has been iteratively refined as damage evidence accumulates, strong-motion data sets enlarge, and insight into response-spectrum features advances. Records obtained after major earthquakes frequently reveal previously unobserved spectral shapes; consequently, identifying these emergent traits and contrasting them with existing spectra constitutes the principal basis for updating code-specified spectra. Whether accounting for local site conditions or for near-source versus far-source effects, the underlying trend is to evolve the design spectrum toward an environment-dependent formulation.

High-quality strong-motion data are the cornerstone of this endeavour. As seismological and ground-motion knowledge deepens, spectral representation must move beyond the conventional “average-and-normalise” paradigm and incorporate additional explanatory factors. Based on regression analysis of large-scale strong-motion records, this study proposes a new spectral shape employing piecewise continuous curves to replace the traditional platform segment averaging approach. This strategy significantly reduces the calibration dispersion caused by platform-type expression (Table 13). Compared with the conventional straight-line platform segment model, the improved model achieves an average reduction of approximately 40% in calibration standard deviation: specifically, the standard deviation decreases from 0.35 to 0.18 for the Zhouqu record (Category Ⅰ site), and from 0.41 to 0.20 for the Luding Jiajun record (Category Ⅱ site), thereby validating the universal applicability of the curved design spectrum across various site categories.

The core advantage of the new spectral shape lies in: eliminating the need for pre-defined parametric models, with the curve morphology entirely driven by statistical regression of real spectra, thereby more accurately mapping the statistical patterns of earthquake acceleration response spectra and achieving high-fidelity fitting between the design spectrum and the original spectrum. The proposed design spectrum calibration model effectively circumvents the systematic bias introduced by the artificial assumption of constant amplification coefficients in the plateau segment inherent to conventional methods, particularly demonstrating superior capability in capturing the actual decay characteristics of response spectra in the medium-to-long period range.However, several aspects of the proposed design spectrum curve remain to be refined for engineering application. Firstly, the normalization issue warrants further investigation: while current codes adopt a single-parameter βmax normalization scheme, the curved features of the new spectral shape necessitate establishing multi-parameter normalization standards to ensure effective compatibility with existing codes. Furthermore, as the proposed method is based on statistical regression, its applicability to regions lacking strong-motion records still requires investigation in conjunction with ground motion prediction equations (GMPE).

## 7. Conclusion

Based on the research focusing on the improvement of design response spectrum calibration methods, the following conclusions are drawn:

(1) Proposal of a new piecewise continuous functional form for design response spectra. Through optimization calibration using genetic algorithms, the proposed spectrum adopts a curved profile in lieu of the conventional horizontal plateau segment, significantly enhancing the fitting accuracy to actual seismic response spectra with a marked reduction in calibration standard deviation compared to traditional approaches.
(2) Establishment of a data-driven calibration methodology for spectral shape parameters. The curved configuration of the proposed method is directly determined from the statistical characteristics of strong-motion records, eliminating the need for pre-specified plateau amplification factors and thereby avoiding systematic errors associated with empirical assumptions inherent in conventional methods. This approach provides a more authentic representation of ground motion spectral characteristics.
(3) Validation of the applicability of the new design spectrum formulation. Verification based on four strong-motion records from the 2008 Wenchuan and 1999 Chi-Chi earthquakes (covering Site Classes I–IV) demonstrates that the improved spectral shape exhibits stable calibration accuracy across diverse site conditions, indicating favorable engineering applicability.
(4) Prospects for future research. The normalization criteria for the new response spectrum curves and the physical interpretation of parameters represent critical issues requiring resolution in subsequent studies to achieve effective integration with current seismic design codes.

## Supporting information

S1 File Data. (ZIP)
