# Supplementary figures and images for: Statistical shape refinement and genetic algorithm calibration of design response spectra based on strong-motion records
Source: PLoS One. 2026 Jul 1;21(7):e0348599. doi: 10.1371/journal.pone.0348599 (PMC13322555; doi:10.1371/journal.pone.0348599)

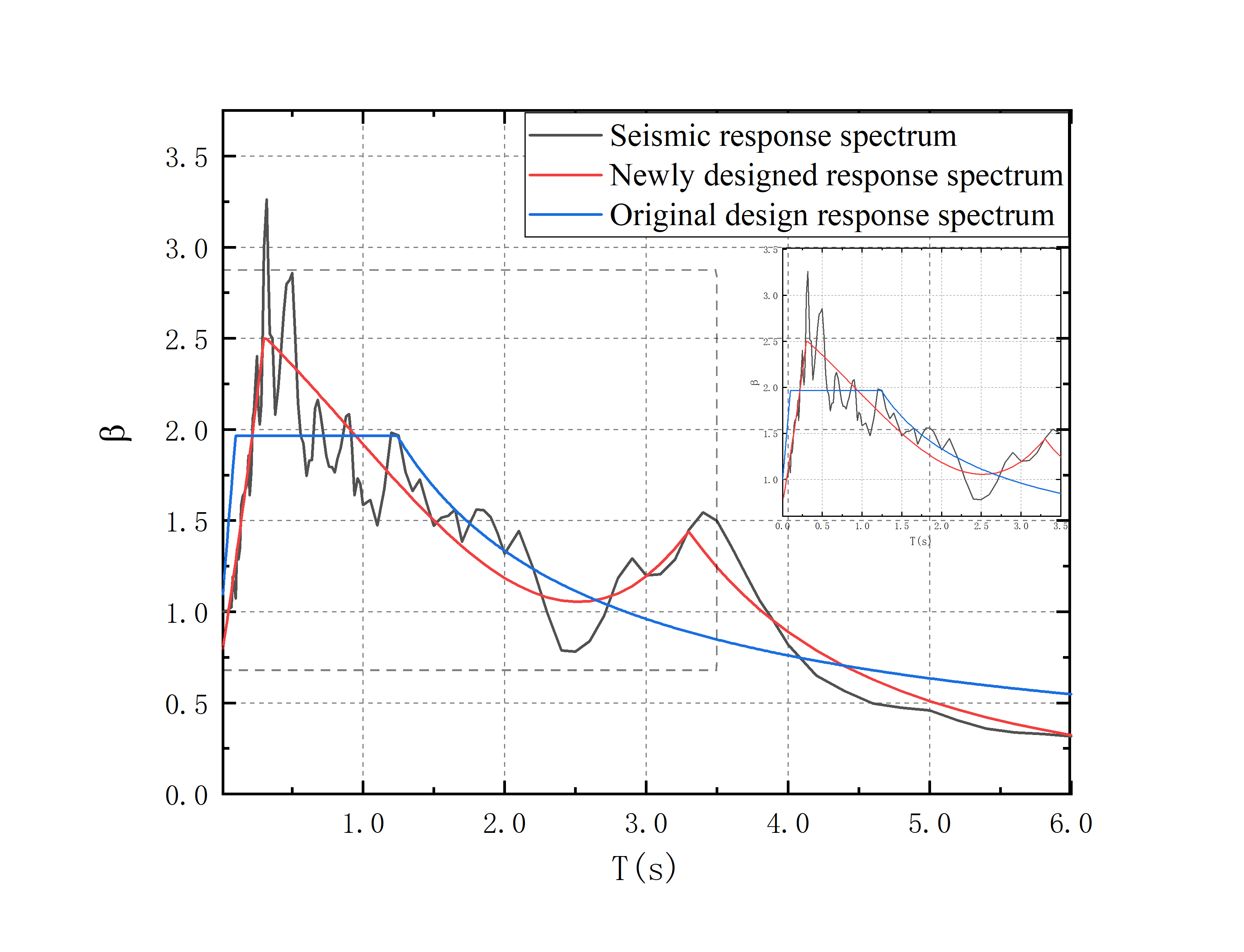

Supplement: S1 File — (ZIP) [file pone.0348599.s001.zip › Data/Comparative Calibration Calculation for Four Site Categories/四类场地集集地震标定对比.jpg]

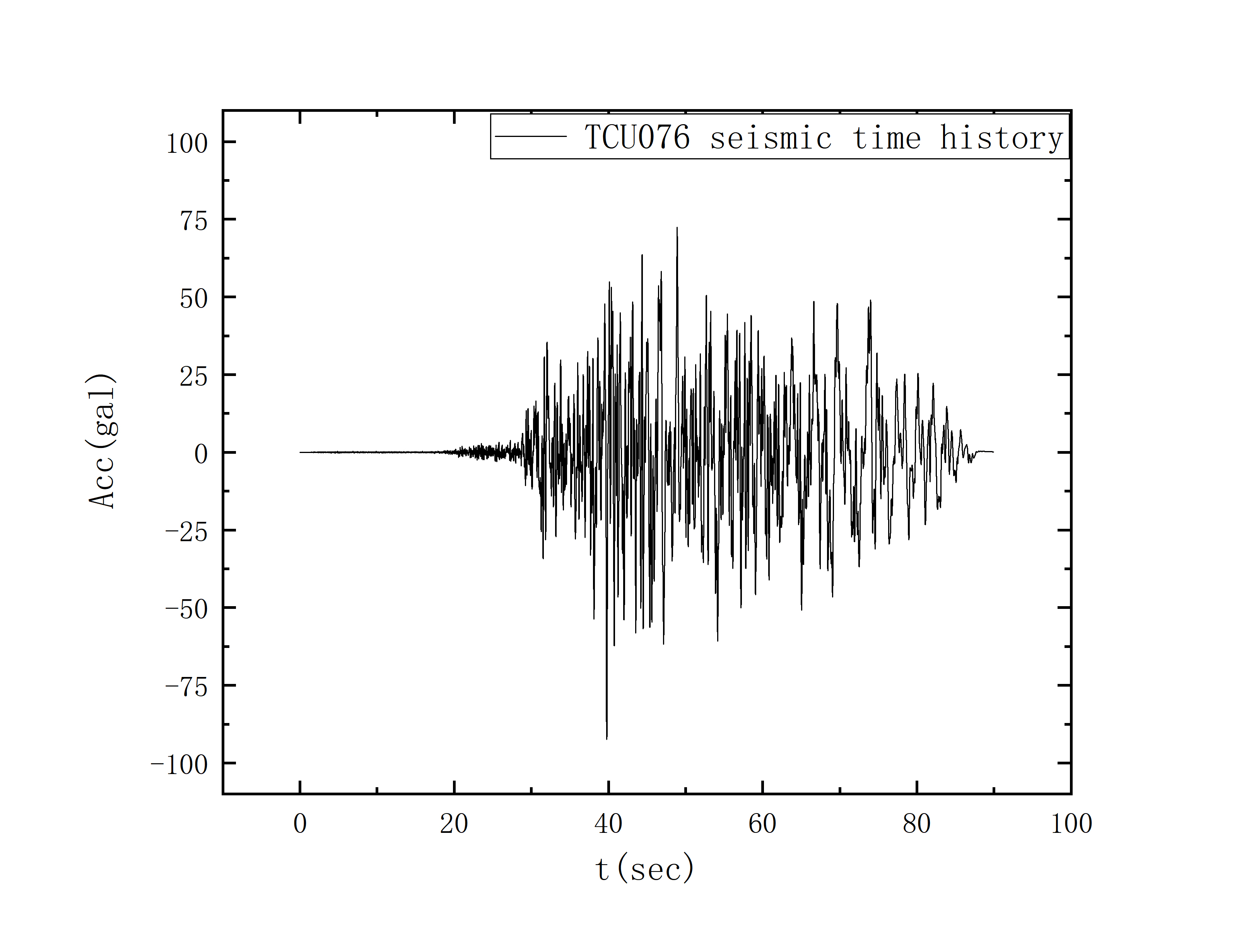

Supplement: S1 File — (ZIP) [file pone.0348599.s001.zip › Data/Comparative Calibration Calculation for Four Site Categories/时程曲线.jpg]
