# Supplementary material for: Statistical shape refinement and genetic algorithm calibration of design response spectra based on strong-motion records
Source: PLoS One. 2026 Jul 1;21(7):e0348599. doi: 10.1371/journal.pone.0348599 (PMC13322555; doi:10.1371/journal.pone.0348599)
Supplement: Attachment — Submitted filename: Response to Reviewers.docx [file pone.0348599.s002.docx]

Response to Reviewers

**Reviewer #1:**

Introduction
The novelty needs to be discussed in comparison with the existing literature, so to support the originality of the paper.
**Many references are only cited, such as 8-11, 22-26. The authors need to discuss them, in order to demonstrate the originality of the paper.
The authors need to describe the app'licability of this study in relation to the ste of the art concept of seismic resilience. Please refer to:
10.1080/15732479.2024.2426036
10.1080/15732479.2025.2591824
10.1016/j.rcns.2025.12.005
10.1016/j.jobe.2025.112282**

**Response to Comment：**Dear reviewers.Thank you for your valuable suggestions.References 8–11 and 22–26 have been supplemented and discussed in the Introduction.

**Revised content is as follows:**Following the work of Newmark and Hall, seismic design codes worldwide have widely adopted this simplified framework, using a horizontal plateau to characterize the medium-frequency spectral shape ^[8-11]^.Major international standards, including Eurocode EN 1998-1, ASCE/SEI 7-10 in the United States, the Turkish Building Seismic Design Code, and the Iranian Code of Practice for Seismic Resistant Design of Buildings, all employ a flat-segment design spectrum. Fundamentally, these are simplified piecewise linear spectra based on statistical averaging. However, this globally prevalent assumption of a horizontal plateau overlooks the pronounced influence of site conditions on response spectrum characteristics and fails to accurately capture the systematic variations in ground motion parameters across different site categories ^[22-26]^.

**Response to Comment：**The four references provided by the reviewers have been carefully studied. The applicability of this research has been discussed from the perspective that Seismic Resilience (SR) has provided a new theoretical framework for expanding traditional response spectrum theory toward whole-life-cycle performance assessment in recent years. These references have been cited as 27–30.

**Revised content is as follows:**Extensive research has demonstrated that site conditions constitute a critical factor governing ground motion parameters. Guo et al. ^[22]^ identified through statistical analysis that site conditions exert a significant influence on the maximum values of design response spectra. Lü and colleagues ^[23]^ proposed recommended values for site influence coefficients based on numerical analyses of site-specific models using data from representative regions, systematically examining the critical issues concerning the impact of site conditions on ground motion parameters. Bo et al. ^[24]^ concluded that soil layer structures significantly affect response spectrum characteristic parameters; specifically, characteristic periods increase with greater overburden thickness. Zhou et al. ^[25]^ conducted statistical analyses of the combined effects of magnitude, epicentral distance, and site conditions on response spectrum characteristics, concluding that buildings designed according to code provisions generally withstand seismic actions exceeding the code-specified levels. Consequently, the range of Site Class II was deliberately expanded as a negotiated compromise. Furthermore, although ground motion selection tools developed by Wang et al. ^[26]^ provide extensive record libraries, they remain constrained by fixed spectral shapes prescribed in design codes for record matching, thereby failing to fundamentally address the systematic discrepancies between code-specified spectra and actual earthquake response spectra. These studies collectively indicate that site dynamic properties play a decisive role in shaping response spectrum morphology; nevertheless, current code spectra continue to employ uniform piecewise linear forms without adequately accounting for spectral shape variations induced by site classification.

Notably, seismic resilience (SR) has emerged in recent years as a central paradigm in earthquake engineering for evaluating structural capabilities to withstand, recover from, and adapt to seismic events, offering a novel theoretical framework that extends traditional response spectrum theory toward whole-life-cycle performance assessment ^[27-30]^. Within this context, Junda and Málaga-Chuquitaype ^[27]^ proposed a stochastic life-cycle assessment (LCA) framework for multi-story cross-laminated timber (CLT) residential buildings that comprehensively accounts for structural time-dependent deterioration and post-earthquake repair requirements. Through nonlinear response history analysis, they demonstrated the substantial influence of long-term structural performance degradation on environmental footprints, underscoring the necessity of incorporating time-varying characteristics into resilience evaluation. This study highlights the critical role of accurate seismic input characterization in quantifying structural damage and subsequent repair demands. Liao and Forcellini ^[28]^ developed an entropy weight-based multi-criteria decision-making methodology to systematically optimize intensity measure (IM) selection for structural seismic resilience assessment. Their findings indicate that peak ground velocity (PGV) and spectral acceleration-based parameters prove most effective in characterizing structural resilience, whereas energy-based metrics tend to underestimate resilience—a discovery with significant implications for the optimization of design spectrum shapes. Forcellini and Kalfas ^[29]^ further established a probabilistic assessment framework quantifying the impact of structural deterioration on seismic resilience. Numerical simulations using OpenSees confirmed that structural degradation substantially diminishes building seismic resilience, particularly regarding recovery velocity, emphasizing the importance of site-specific ground motion characterization for accurately predicting structural time-varying responses. Additionally, Forcellini and Kalfas ^[30]^ proposed an integrated multi-hazard (seismic-fire) risk assessment framework for reinforced concrete buildings that incorporates environmental deterioration effects including chloride-induced corrosion and carbonation. Their work revealed that neglecting continuous structural degradation and cumulative seismic damage significantly increases post-earthquake fire risk, imposing more stringent requirements on design spectra to account for multi-hazard coupling and whole-life-cycle performance evolution. These state-of-the-art studies in seismic resilience collectively demonstrate that modern seismic assessment has evolved from purely pre-earthquake resistance analysis toward a comprehensive system encompassing structural whole-life-cycle performance evolution, multi-hazard coupling effects, and recovery capability quantification. This paradigm emphasizes the deep integration of time-varying reliability, multi-criteria decision-making, and dynamic risk management, presenting new challenges and improvement requirements for traditional simplified design spectra based on statistical averaging.

In contrast to the aforementioned studies, and distinct from existing literature that focuses exclusively on the influence of site conditions on characteristic response spectrum parameters—such as maximum spectral values^[22]^ or specific frequency band features ^[23-25]^.Rather than adopting the code-specified spectral shape, this study abandons any predefined functional form and, within a coupled seismic-environment and site-condition framework, employs the frequency-domain characteristics of strong-motion acceleration response spectra as the sole basis for constructing the design spectrum.

**Section 1
Reproducibility is fundamental in scientific papers and the authors need to discuss the choices and approaches they use. For example, the authors need to describe this part: "to eliminate (...) averaged".**

**Response to Comment：**Dear reviewers.Thank you for your valuable suggestions.Here, a discussion on the normalization of response spectra to eliminate the influence of ground motion peak values on seismic acceleration response spectra has been added, clarifying the purpose and significance of normalized response spectrum calculations.

**Revised content is as follows:**To eliminate the influence of the ground-motion amplitude on the seismic acceleration response spectra, each spectrum was normalized by the peak ground acceleration (PGA) of the corresponding record, yielding the dynamic amplification coefficient (DAC) spectrum. DAC spectra from all records within each site class were subsequently averaged.This study employs a site-classification-based grouping and averaging strategy to statistically analyze dynamic amplification coefficient (DAC) spectra across four site categories (Classes I, II, III, and IV). The selection of this methodology is motivated by the following three considerations:

(1) Statistical stability and outlier control: Geometric mean averaging of a large number of records effectively filters event-specific high-frequency oscillations and anomalous peaks, extracting a statistically stable expected spectral shape that reduces the sensitivity of design spectra to sporadic extrema. Compared with arithmetic mean, geometric mean more effectively suppresses the dominant influence of high-amplitude outlier records and better aligns with the statistical distribution characteristics of ground motion parameters [31].

(2) Extraction of intrinsic site characteristics: Averaging records within the same site category suppresses case-specific deviations arising from random factors such as topographic effects and source-path variations, highlighting the intrinsic "site–spectrum" relationship and thereby providing physically meaningful representative spectral shapes for different site classes.

(3) Reproducibility assurance: To ensure the reproducibility of research findings, this study strictly adheres to the following standardized procedures: (i) Record selection criteria: Only free-field strong-motion records with complete data, satisfying the magnitude and peak acceleration screening criteria specified previously, are utilized; (ii) Data processing protocol: All records undergo consistent filtering and baseline correction; (iii) Response spectrum calculation: Absolute acceleration response spectra are computed with 5% critical damping and a uniform time step of 0.01 s; (iv) Grouping basis: Site classifications are strictly assigned according to the categorization standards described above, ensuring consistency with engineering practice.

**Section 2
I suggest to make a unique table to show all the performed cases.The authors need to expand this part: "Consequently (...) classes"**.

**Response to Comment：**Dear reviewers.Thank you for your valuable suggestions.Table 8 summarizes the maximum R² values and corresponding regression models for the four site classes. The discussion in the "Consequently (...) classes" section has been supplemented and elaborated.

**Revised content is as follows:**Consequently, equation (2) is uniformly employed for sites I–III, and equation (3) for site IV, thereby fully defining the seismic acceleration response spectra for all four site classes.This piecewise model framework ensures continuity and consistency between the high-frequency and medium-frequency ranges across all four site categories, while fully accounting for the divergent attenuation characteristics in the low-frequency range induced by varying site conditions. This approach achieves a unified balance between theoretical rigor and engineering practicality.

**Section 3
Title: I recommend to avoid the word "determination", it is too generic**.

**Response to Comment：**Dear reviewers.Thank you for your valuable suggestions.The subsection title in Chapter 3 has been revised to "Calibration of Response Spectrum Shape Control Parameters".

**Section 4
The authors need to discuss the importance of the site effects in the definition of the response spectrum curves. Whan the bedrock input are necessary to be considered? Please expand this part.
The authors wrote that the calibration error analysis demonstrated that the standard deviation is lower than that of the original version of the spectra. They need to refer to existing case studies.**

**Response to Comment：**Dear reviewers.Thank you for your valuable suggestions.In the comparative analysis of the calibration of new and old design response spectrum curves in Chapter 4, the discussion on the importance of site effects in defining response spectrum curves has been supplemented. Meanwhile, considerations regarding bedrock input in the calculations have also been provided.

**Revised content is as follows:**Based on the mean dynamic amplification factor spectra for four site categories, this study establishes the expression form of the design spectrum through numerical analysis of site models. Site effects constitute the core factor in defining design response spectrum curves, reflecting the alteration of spectral characteristics during seismic wave propagation from bedrock to ground surface due to soil filtering and resonance effects. Distinct site categories exhibit markedly different dynamic amplification properties: soft soil sites (Categories Ⅲ and Ⅳ) typically demonstrate longer characteristic periods and greater amplification of long-period components, whereas stiff sites (Categories Ⅰ and Ⅱ) exhibit short-period, high-amplitude amplification characteristics. Consequently, the modulating effect of site conditions on ground motion spectral shape must be fully accounted for during design response spectrum calibration.

Regarding the consideration of bedrock input, it is primarily applicable to the following scenarios: (1) When a definitive engineering bedrock surface exists beneath the project site with a bedrock wave velocity exceeding 500 m/s, the bedrock should be adopted as the reference plane for seismic motion input to accurately characterize the amplification effect of soil layers on seismic waves; (2) For sites with deep overburden deposits or complex soil layer structures, bedrock input is required to conduct one-dimensional or two-dimensional site seismic response analyses, thereby obtaining surface design ground motion parameters, followed by site adjustment through site classification. The Category Ⅰ–Ⅳ site records selected in this study all account for the influence of site conditions on ground motion, wherein Category Ⅰ sites approximate bedrock conditions, while Categories Ⅱ–Ⅳ sites exhibit varying degrees of soil amplification effects.

**Response to Comment：**Regarding the calibration error analysis, relevant reference cases have been cited for illustration. The error analysis Table 12 has been provided by calculating the standard deviation to demonstrate that the standard deviation is lower than that of the original version spectrum.

**Revised content is as follows:** Table 12 presents the error analysis for the two calibration methods. The calibration error analysis indicates that the standard deviation of the improved design response spectrum is significantly lower than that of the original design response spectrum, and its curve more accurately reflects the spectral characteristics of the seismic response spectrum. This result is consistent with Chopra (2012) regarding curved platform segments of design spectra providing better fitting to actual response spectra^[44]^, and also aligns with the analysis results of Xie Lili et al. (2012) for Wenchuan earthquake records, thereby validating the importance of the improved spectral shape in engineering applications^[45]^.

**Conclusions
The conclusions commmonly describe the findings, the possible applications and future work. Two different sections should me used: Discussion and Conclusions.**

**Response to Comment：**Dear reviewers.Thank you for your valuable suggestions.The Conclusion section has been revised and divided into two separate chapters: Discussion and Conclusions.

**Revised content is as follows:**

Discussion

The calibration of design response spectra should, while retaining engineering practicality, fully reflect the contemporary understanding of strong ground-motion characteristics in earthquake engineering. Historical review of seismic design codes shows that the design spectrum has been iteratively refined as damage evidence accumulates, strong-motion data sets enlarge, and insight into response-spectrum features advances. Records obtained after major earthquakes frequently reveal previously unobserved spectral shapes; consequently, identifying these emergent traits and contrasting them with existing spectra constitutes the principal basis for updating code-specified spectra. Whether accounting for local site conditions or for near-source versus far-source effects, the underlying trend is to evolve the design spectrum toward an environment-dependent formulation.

High-quality strong-motion data are the cornerstone of this endeavour. As seismological and ground-motion knowledge deepens, spectral representation must move beyond the conventional "average-and-normalise" paradigm and incorporate additional explanatory factors. Based on regression analysis of large-scale strong-motion records, this study proposes a new spectral shape employing piecewise continuous curves to replace the traditional platform segment averaging approach. This strategy significantly reduces the calibration dispersion caused by platform-type expression (Table 12). Compared with the conventional straight-line platform segment model, the improved model achieves an average reduction of approximately 40% in calibration standard deviation: specifically, the standard deviation decreases from 0.35 to 0.18 for the Zhouqu record (Category Ⅰ site), and from 0.41 to 0.20 for the Luding Jiajun record (Category Ⅱ site), thereby validating the universal applicability of the curved design spectrum across various site categories.

The core advantage of the new spectral shape lies in: eliminating the need for pre-defined parametric models, with the curve morphology entirely driven by statistical regression of real spectra, thereby more accurately mapping the statistical patterns of earthquake acceleration response spectra and achieving high-fidelity fitting between the design spectrum and the original spectrum. The proposed design spectrum calibration model effectively circumvents the systematic bias introduced by the artificial assumption of constant amplification coefficients in the plateau segment inherent to conventional methods, particularly demonstrating superior capability in capturing the actual decay characteristics of response spectra in the medium-to-long period range.However, several aspects of the proposed design spectrum curve remain to be refined for engineering application. Firstly, the normalization issue warrants further investigation: while current codes adopt a single-parameter βmax normalization scheme, the curved features of the new spectral shape necessitate establishing multi-parameter normalization standards to ensure effective compatibility with existing codes. Furthermore, as the proposed method is based on statistical regression, its applicability to regions lacking strong-motion records still requires investigation in conjunction with ground motion prediction equations (GMPE).

Conclusion

Based on the research focusing on the improvement of design response spectrum calibration methods, the following conclusions are drawn:

(1) Proposal of a new piecewise continuous functional form for design response spectra. Through optimization calibration using genetic algorithms, the proposed spectrum adopts a curved profile in lieu of the conventional horizontal plateau segment, significantly enhancing the fitting accuracy to actual seismic response spectra with a marked reduction in calibration standard deviation compared to traditional approaches.

(2) Establishment of a data-driven calibration methodology for spectral shape parameters. The curved configuration of the proposed method is directly determined from the statistical characteristics of strong-motion records, eliminating the need for pre-specified plateau amplification factors and thereby avoiding systematic errors associated with empirical assumptions inherent in conventional methods. This approach provides a more authentic representation of ground motion spectral characteristics.

(3) Validation of the applicability of the new design spectrum formulation. Verification based on four strong-motion records from the 2008 Wenchuan and 1999 Chi-Chi earthquakes (covering Site Classes I–IV) demonstrates that the improved spectral shape exhibits stable calibration accuracy across diverse site conditions, indicating favorable engineering applicability.

(4) Prospects for future research. The normalization criteria for the new response spectrum curves and the physical interpretation of parameters represent critical issues requiring resolution in subsequent studies to achieve effective integration with current seismic design codes.

**Reviewer #2:**

**1- How different model parameters of GA could affect the results?**

**Response to Comment：**Dear reviewers.Thank you for your valuable suggestions.The influence of different model parameters of the Genetic Algorithm (GA) on the results has been supplemented in Chapter 3.

**Revised content is as follows:**

**C: Genetic algorithm parameter optimization and sensitivity analysis**

To optimize and calibrate the nine spectral shape control parameters in the aforementioned models, this study introduces the Genetic Algorithm (GA) [36]. GA is an adaptive global optimization algorithm that simulates biological inheritance and evolutionary mechanisms in nature. Owing to its high efficiency, practicality, and strong robustness, GA has been extensively applied to nonlinear optimization, multimodal function optimization, and multi-objective optimization problems [36-38]. Specifically, Equation (4) represents the response spectrum calibration model for Site Classes I, II, and III, whereas Equation (5) corresponds to the model for Site Class IV. Drawing upon the hybrid encoding strategy proposed by Alimoradi et al. [添加39], this study employs a mixed binary-real encoding scheme: period parameters (T₀, Tg) are encoded as real numbers to ensure precision, while spectral shape coefficients (a₁–a₆, γ) are binary-encoded to facilitate genetic operations.

**D: **Design of Response Spectrum Calibration and Dynamic Update Mechanism****

The genetic algorithm is employed to calibrate the design response spectrum, with the objective of minimizing the standard deviation Q to determine the corner periods ($T_{0}、T_{g}$), the spectral decay exponent $\gamma$, and other spectral shape control parameters ($a_{1}\sim a_{6}$). When the standard deviation Q reaches its minimum value, the corresponding parameters are regarded as the optimal spectral shape control parameters. The calculation formulas for the standard deviation Q are given by Equations (6) and (7).With the expansion of strong-motion data, dynamic updating of existing design spectra can be implemented subsequently through rolling average or weighted average approaches, without the need to reconstruct the entire curve. The differential magnitude between the old and new spectra can be attributed to incremental changes in the averaged sample, thereby providing traceable and quantifiable scientific evidence for code revision. Regarding the specific computational procedures of the genetic algorithm for target response spectrum and characteristic parameter calibration, refer to references [40-41]; further elaboration is omitted here.

**2- There are some new works which should be read and cited well in the text, for example
-Ground motion records selection based on scalar frequency-content parameters
- Selection and scaling of spectrum-compatible ground motion records using hybrid coded genetic algorithms**

**Response to Comment：**Dear reviewers.Thank you for your valuable suggestions.The contributions of the authors of these two references have been thoroughly reviewed and analyzed, with their research findings appropriately cited and integrated into the current study.

**Revised content is as follows:**

An excessively small population size tends to cause premature convergence and entrapment in local optima (eg, Tg deviation > 15%) [39]; an excessively large population size increases computational cost but enhances the probability of locating the global optimum. Empirical tests indicate that 200 individuals strike a balance between computational efficiency and convergence reliability.（Table 9）.

Inspired by the scalar frequency-content parameter concept presented by Yaghmaei-Sabegh and Jodat-Saeidabad in Review Reference [37], this study incorporates the mean period (Tm) and average spectral period (Tavg) as criteria for ground motion clustering during the initial population generation stage of the genetic algorithm. By ensuring a uniform spatial distribution of the initial population within the frequency-content domain, the proposed approach substantially improves convergence efficiency, achieving an average reduction of approximately 25% in evolutionary generations.
