# Supplementary material for: Statistical shape refinement and genetic algorithm calibration of design response spectra based on strong-motion records
Source: PLoS One. 2026 Jul 1;21(7):e0348599. doi: 10.1371/journal.pone.0348599 (PMC13322555; doi:10.1371/journal.pone.0348599)
Supplement: Peer review history

# Peer review: Decision Letter 0

**DOI:** 10.1371/journal.pone.0348599.r001

**Authors:** Alessandro Pluchino

8 Feb 2026

PONE-D-26-02245

Statistical Shape Refinement and Genetic Algorithm Calibration of Design Response Spectra Based on Strong-Motion Records

PLOS One

Dear Dr. Han,

Thank you for submitting your manuscript to PLOS ONE. After careful consideration, we feel that it has merit but does not fully meet PLOS ONE’s publication criteria as it currently stands. Therefore, we invite you to submit a revised version of the manuscript that addresses the points raised during the review process.

Please submit your revised manuscript by Mar 25 2026 11:59PM. If you will need more time than this to complete your revisions, please reply to this message or contact the journal office at plosone@plos.org. When you're ready to submit your revision, log on to https://www.editorialmanager.com/pone/ and select the 'Submissions Needing Revision' folder to locate your manuscript file.

Please include the following items when submitting your revised manuscript:

- A letter that responds to each point raised by the academic editor and reviewer(s). You should upload this letter as a separate file labeled 'Response to Reviewers'.
- A marked-up copy of your manuscript that highlights changes made to the original version. You should upload this as a separate file labeled 'Revised Manuscript with Track Changes'.
- An unmarked version of your revised paper without tracked changes. You should upload this as a separate file labeled 'Manuscript'.

If you would like to make changes to your financial disclosure, please include your updated statement in your cover letter. Guidelines for resubmitting your figure files are available below the reviewer comments at the end of this letter.

If applicable, we recommend that you deposit your laboratory protocols in protocols.io to enhance the reproducibility of your results. Protocols.io assigns your protocol its own identifier (DOI) so that it can be cited independently in the future. For instructions see: https://journals.plos.org/plosone/s/submission-guidelines#loc-laboratory-protocols. Additionally, PLOS ONE offers an option for publishing peer-reviewed Lab Protocol articles, which describe protocols hosted on protocols.io. Read more information on sharing protocols at https://plos.org/protocols?utm_medium=editorial-email&utm_source=authorletters&utm_campaign=protocols.

We look forward to receiving your revised manuscript.

Kind regards,

Alessandro Pluchino

Academic Editor

PLOS One

**Journal Requirements:**

When submitting your revision, we need you to address these additional requirements.

1. Please ensure that your manuscript meets PLOS ONE's style requirements, including those for file naming. The PLOS ONE style templates can be found at

https://journals.plos.org/plosone/s/file?id=wjVg/PLOSOne_formatting_sample_main_body.pdf and

https://journals.plos.org/plosone/s/file?id=ba62/PLOSOne_formatting_sample_title_authors_affiliations.pdf

2. Please note that PLOS One has specific guidelines on code sharing for submissions in which author-generated code underpins the findings in the manuscript. In these cases, we expect all author-generated code to be made available without restrictions upon publication of the work. Please review our guidelines at https://journals.plos.org/plosone/s/materials-and-software-sharing#loc-sharing-code and ensure that your code is shared in a way that follows best practice and facilitates reproducibility and reuse.

3. Please update your submission to use the PLOS LaTeX template. The template and more information on our requirements for LaTeX submissions can be found at http://journals.plos.org/plosone/s/latex.

4. We note that the grant information you provided in the ‘Funding Information’ and ‘Financial Disclosure’ sections do not match.

When you resubmit, please ensure that you provide the correct grant numbers for the awards you received for your study in the ‘Funding Information’ section.

5. Please note that funding information should not appear in any section or other areas of your manuscript. We will only publish funding information present in the Funding Statement section of the online submission form. Please remove any funding-related text from the manuscript.

6. We note that your Data Availability Statement is currently as follows:

“All relevant data are within the manuscript and its Supporting Information files.”

Please confirm at this time whether or not your submission contains all raw data required to replicate the results of your study. Authors must share the “minimal data set” for their submission. PLOS defines the minimal data set to consist of the data required to replicate all study findings reported in the article, as well as related metadata and methods (https://journals.plos.org/plosone/s/data-availability#loc-minimal-data-set-definition).

For example, authors should submit the following data:

- The values behind the means, standard deviations and other measures reported;

- The values used to build graphs;

- The points extracted from images for analysis.

Authors do not need to submit their entire data set if only a portion of the data was used in the reported study.

If your submission does not contain these data, please either upload them as Supporting Information files or deposit them to a stable, public repository and provide us with the relevant URLs, DOIs, or accession numbers. For a list of recommended repositories, please see https://journals.plos.org/plosone/s/recommended-repositories.

If there are ethical or legal restrictions on sharing a de-identified data set, please explain them in detail (e.g., data contain potentially sensitive information, data are owned by a third-party organization, etc.) and who has imposed them (e.g., an ethics committee). Please also provide contact information for a data access committee, ethics committee, or other institutional body to which data requests may be sent. If data are owned by a third party, please indicate how others may request data access.

7. We note you have included a table to which you do not refer in the text of your manuscript. Please ensure that you refer to Tables 11 and 12 in your text; if accepted, production will need this reference to link the reader to the Table.

8. If the reviewer comments include a recommendation to cite specific previously published works, please review and evaluate these publications to determine whether they are relevant and should be cited. There is no requirement to cite these works unless the editor has indicated otherwise.

[Note: HTML markup is below. Please do not edit.]

Reviewers' comments:

Reviewer's Responses to Questions

**Comments to the Author**

1. Is the manuscript technically sound, and do the data support the conclusions?

The manuscript must describe a technically sound piece of scientific research with data that supports the conclusions. Experiments must have been conducted rigorously, with appropriate controls, replication, and sample sizes. The conclusions must be drawn appropriately based on the data presented.

Reviewer #1: Yes

Reviewer #2: Yes

**********

2. Has the statistical analysis been performed appropriately and rigorously?

Reviewer #1: Yes

Reviewer #2: Yes

**********

3. Have the authors made all data underlying the findings in their manuscript fully available?

The PLOS Data policy requires authors to make all data underlying the findings described in their manuscript fully available without restriction, with rare exception (please refer to the Data Availability Statement in the manuscript PDF file). The data should be provided as part of the manuscript or its supporting information, or deposited to a public repository. For example, in addition to summary statistics, the data points behind means, medians and variance measures should be available. If there are restrictions on publicly sharing data—e.g. participant privacy or use of data from a third party—those must be specified.

Reviewer #1: Yes

Reviewer #2: Yes

**********

4. Is the manuscript presented in an intelligible fashion and written in standard English?

PLOS ONE does not copyedit accepted manuscripts, so the language in submitted articles must be clear, correct, and unambiguous. Any typographical or grammatical errors should be corrected at revision, so please note any specific errors here.

Reviewer #1: Yes

Reviewer #2: Yes

**********

5. Review Comments to the Author

Please use the space provided to explain your answers to the questions above. You may also include additional comments for the author, including concerns about dual publication, research ethics, or publication ethics. (Please upload your review as an attachment if it exceeds 20,000 characters)

Reviewer #1: he paper investigates an interesting topic. English is fine and the methodology is pertinent. However, some issues need to be considered.

Introduction

The novelty needs to be discussed in comparison with the existing literature, so to support the originality of the paper.

Many references are only cited, such as 8-11, 22-26. The authors need to discuss them, in order to demonstrate the originality of the paper.

The authors need to describe the app'licability of this study in relation to the ste of the art concept of seismic resilience. Please refer to:

10.1080/15732479.2024.2426036

10.1080/15732479.2025.2591824

10.1016/j.rcns.2025.12.005

10.1016/j.jobe.2025.112282

Section 1

Reproducibility is fundamental in scientific papers and the authors need to discuss the choices and approaches they use.

For example, the authors need to describe this part: "to eliminate (...) averaged".

Section 2

I suggest to make a unique table to show all the performed cases.

The authors need to expand this part: "Consequently (...) classes".

Section 3

Title: I recommend to avoid the word "determination", it is too generic.

Section 4

The authors need to discuss the importance of the site effects in the definition of the response spectrum curves. Whan the bedrock input are necessary to be considered? Please expand this part.

The authors wrote that the calibration error analysis demonstrated that the standard deviation is lower than that of the original version of the spectra. They need to refer to existing case studies.

Conclusions

The conclusions commmonly describe the findings, the possible applications and future work. Two different sections should me used: Discussion and Conclusions.

Reviewer #2: This is a relatively good work and could be useful for readers, however the following comments should be considered

1- How different model parameters of GA could affect the results?

2- There are some new works which should be read and cited well in the text, for example

-Ground motion records selection based on scalar frequency-content parameters

- Selection and scaling of spectrum-compatible ground motion records using hybrid coded genetic algorithms

**********

6. PLOS authors have the option to publish the peer review history of their article (what does this mean?). If published, this will include your full peer review and any attached files.

If you choose “no”, your identity will remain anonymous but your review may still be made public.

**Do you want your identity to be public for this peer review?**  For information about this choice, including consent withdrawal, please see our Privacy Policy.

Reviewer #1: No

Reviewer #2: No

**********

[NOTE: If reviewer comments were submitted as an attachment file, they will be attached to this email and accessible via the submission site. Please log into your account, locate the manuscript record, and check for the action link "View Attachments". If this link does not appear, there are no attachment files.]

To ensure your figures meet our technical requirements, please review our figure guidelines: https://journals.plos.org/plosone/s/figures

You may also use PLOS’s free figure tool, NAAS, to help you prepare publication quality figures: https://journals.plos.org/plosone/s/figures#loc-tools-for-figure-preparation.

NAAS will assess whether your figures meet our technical requirements by comparing each figure against our figure specifications.

# Author comment: Author response to Decision Letter 1

**DOI:** 10.1371/journal.pone.0348599.r002

2 Apr 2026

Dear Dr. Alessandro Pluchino,

Academic Editor, PLOS ONE

I am pleased to inform you that I have completed the comprehensive revision of our manuscript, addressing all comments and suggestions raised by the reviewers in accordance with PLOS ONE editorial guidelines. The following documents have been submitted through the online system:

Clean version of the revised manuscript

Revised manuscript with tracked changes

Point-by-point response to reviewers

Thank you for your time and consideration. I look forward to your decision.

Sincerely,

Response to Reviewers

Reviewer #1:

Introduction

The novelty needs to be discussed in comparison with the existing literature, so to support the originality of the paper.

Many references are only cited, such as 8-11, 22-26. The authors need to discuss them, in order to demonstrate the originality of the paper.

The authors need to describe the app'licability of this study in relation to the ste of the art concept of seismic resilience. Please refer to:

10.1080/15732479.2024.2426036

10.1080/15732479.2025.2591824

10.1016/j.rcns.2025.12.005

10.1016/j.jobe.2025.112282

Response to Comment：Dear reviewers.Thank you for your valuable suggestions.References 8–11 and 22–26 have been supplemented and discussed in the Introduction.

Revised content is as follows:Following the work of Newmark and Hall, seismic design codes worldwide have widely adopted this simplified framework, using a horizontal plateau to characterize the medium-frequency spectral shape [8-11].Major international standards, including Eurocode EN 1998-1, ASCE/SEI 7-10 in the United States, the Turkish Building Seismic Design Code, and the Iranian Code of Practice for Seismic Resistant Design of Buildings, all employ a flat-segment design spectrum. Fundamentally, these are simplified piecewise linear spectra based on statistical averaging. However, this globally prevalent assumption of a horizontal plateau overlooks the pronounced influence of site conditions on response spectrum characteristics and fails to accurately capture the systematic variations in ground motion parameters across different site categories [22-26].

Response to Comment：The four references provided by the reviewers have been carefully studied. The applicability of this research has been discussed from the perspective that Seismic Resilience (SR) has provided a new theoretical framework for expanding traditional response spectrum theory toward whole-life-cycle performance assessment in recent years. These references have been cited as 27–30.

Revised content is as follows:Extensive research has demonstrated that site conditions constitute a critical factor governing ground motion parameters. Guo et al. [22] identified through statistical analysis that site conditions exert a significant influence on the maximum values of design response spectra. Lü and colleagues [23] proposed recommended values for site influence coefficients based on numerical analyses of site-specific models using data from representative regions, systematically examining the critical issues concerning the impact of site conditions on ground motion parameters. Bo et al. [24] concluded that soil layer structures significantly affect response spectrum characteristic parameters; specifically, characteristic periods increase with greater overburden thickness. Zhou et al. [25] conducted statistical analyses of the combined effects of magnitude, epicentral distance, and site conditions on response spectrum characteristics, concluding that buildings designed according to code provisions generally withstand seismic actions exceeding the code-specified levels. Consequently, the range of Site Class II was deliberately expanded as a negotiated compromise. Furthermore, although ground motion selection tools developed by Wang et al. [26] provide extensive record libraries, they remain constrained by fixed spectral shapes prescribed in design codes for record matching, thereby failing to fundamentally address the systematic discrepancies between code-specified spectra and actual earthquake response spectra. These studies collectively indicate that site dynamic properties play a decisive role in shaping response spectrum morphology; nevertheless, current code spectra continue to employ uniform piecewise linear forms without adequately accounting for spectral shape variations induced by site classification.

Notably, seismic resilience (SR) has emerged in recent years as a central paradigm in earthquake engineering for evaluating structural capabilities to withstand, recover from, and adapt to seismic events, offering a novel theoretical framework that extends traditional response spectrum theory toward whole-life-cycle performance assessment [27-30]. Within this context, Junda and Málaga-Chuquitaype [27] proposed a stochastic life-cycle assessment (LCA) framework for multi-story cross-laminated timber (CLT) residential buildings that comprehensively accounts for structural time-dependent deterioration and post-earthquake repair requirements. Through nonlinear response history analysis, they demonstrated the substantial influence of long-term structural performance degradation on environmental footprints, underscoring the necessity of incorporating time-varying characteristics into resilience evaluation. This study highlights the critical role of accurate seismic input characterization in quantifying structural damage and subsequent repair demands. Liao and Forcellini [28] developed an entropy weight-based multi-criteria decision-making methodology to systematically optimize intensity measure (IM) selection for structural seismic resilience assessment. Their findings indicate that peak ground velocity (PGV) and spectral acceleration-based parameters prove most effective in characterizing structural resilience, whereas energy-based metrics tend to underestimate resilience—a discovery with significant implications for the optimization of design spectrum shapes. Forcellini and Kalfas [29] further established a probabilistic assessment framework quantifying the impact of structural deterioration on seismic resilience. Numerical simulations using OpenSees confirmed that structural degradation substantially diminishes building seismic resilience, particularly regarding recovery velocity, emphasizing the importance of site-specific ground motion characterization for accurately predicting structural time-varying responses. Additionally, Forcellini and Kalfas [30] proposed an integrated multi-hazard (seismic-fire) risk assessment framework for reinforced concrete buildings that incorporates environmental deterioration effects including chloride-induced corrosion and carbonation. Their work revealed that neglecting continuous structural degradation and cumulative seismic damage significantly increases post-earthquake fire risk, imposing more stringent requirements on design spectra to account for multi-hazard coupling and whole-life-cycle performance evolution. These state-of-the-art studies in seismic resilience collectively demonstrate that modern seismic assessment has evolved from purely pre-earthquake resistance analysis toward a comprehensive system encompassing structural whole-life-cycle performance evolution, multi-hazard coupling effects, and recovery capability quantification. This paradigm emphasizes the deep integration of time-varying reliability, multi-criteria decision-making, and dynamic risk management, presenting new challenges and improvement requirements for traditional simplified design spectra based on statistical averaging.

In contrast to the aforementioned studies, and distinct from existing literature that focuses exclusively on the influence of site conditions on characteristic response spectrum parameters—such as maximum spectral values[22] or specific frequency band features [23-25].Rather than adopting the code-specified spectral shape, this study abandons any predefined functional form and, within a coupled seismic-environment and site-condition framework, employs the frequency-domain characteristics of strong-motion acceleration response spectra as the sole basis for constructing the design spectrum.

Section 1

Reproducibility is fundamental in scientific papers and the authors need to discuss the choices and approaches they use. For example, the authors need to describe this part: "to eliminate (...) averaged".

Response to Comment：Dear reviewers.Thank you for your valuable suggestions.Here, a discussion on the normalization of response spectra to eliminate the influence of ground motion peak values on seismic acceleration response spectra has been added, clarifying the purpose and significance of normalized response spectrum calculations.

Revised content is as follows:To eliminate the influence of the ground-motion amplitude on the seismic acceleration response spectra, each spectrum was normalized by the peak ground acceleration (PGA) of the corresponding record, yielding the dynamic amplification coefficient (DAC) spectrum. DAC spectra from all records within each site class were subsequently averaged.This study employs a site-classification-based grouping and averaging strategy to statistically analyze dynamic amplification coefficient (DAC) spectra across four site categories (Classes I, II, III, and IV). The selection of this methodology is motivated by the following three considerations:

(1) Statistical stability and outlier control: Geometric mean averaging of a large number of records effectively filters event-specific high-frequency oscillations and anomalous peaks, extracting a statistically stable expected spectral shape that reduces the sensitivity of design spectra to sporadic extrema. Compared with arithmetic mean, geometric mean more effectively suppresses the dominant influence of high-amplitude outlier records and better aligns with the statistical distribution characteristics of ground motion parameters [31].

(2) Extraction of intrinsic site characteristics: Averaging records within the same site category suppresses case-specific deviations arising from random factors such as topographic effects and source-path variations, highlighting the intrinsic "site–spectrum" relationship and thereby providing physically meaningful representative spectral shapes for different site classes.

(3) Reproducibility assurance: To ensure the reproducibility of research findings, this study strictly adheres to the following standardized procedures: (i) Record selection criteria: Only free-field strong-motion records with complete data, satisfying the magnitude and peak acceleration screening criteria specified previously, are utilized; (ii) Data processing protocol: All records undergo consistent filtering and baseline correction; (iii) Response spectrum calculation: Absolute acceleration response spectra are computed with 5% critical damping and a uniform time step of 0.01 s; (iv) Grouping basis: Site classifications are strictly assigned according to the categorization standards described above, ensuring consistency with engineering practice.

Section 2

I suggest to make a unique table to show all the performed cases.The authors need to expand this part: "Consequently (...) classes".

Response to Comment：Dear reviewers.Thank you for your valuable suggestions.Table 8 summarizes the maximum R² values and corresponding regression models for the four site classes. The discussion in the "Consequently (...) classes" section has been supplemented and elaborated.

Revised content is as follows:Consequently, equation (2) is uniformly employed for sites I–III, and equation (3) for site IV, thereby fully defining the seismic acceleration response spectra for all four site classes.This piecewise model framework ensures continuity and consistency between the high-frequency and medium-frequency ranges across all four site categories, while fully accounting for the divergent attenuation characteristics in the low-frequency range induced by varying site conditions. This approach achieves a unified balance between theoretical rigor and engineering practicality.

Section 3

Title: I recommend to avoid the word "determination", it is too generic.

Response to Comment：Dear reviewers.Thank you for your valuable suggestions.The subsection title in Chapter 3 has been revised to "Calibration of Response Spectrum Shape Control Parameters".

Section 4

The authors need to discuss the importance of the site effects in the definition of the response spectrum curves. Whan the bedrock input are necessary to be considered? Please expand this part.

The authors wrote that the calibration error analysis demonstrated that the standard deviation is lower than that of the original version of the spectra. They need to refer to existing case studies.

Response to Comment：Dear reviewers.Thank you for your valuable suggestions.In the comparative analysis of the calibration of new and old design response spectrum curves in Chapter 4, the discussion on the importance of site effects in defining response spectrum curves has been supplemented. Meanwhile, considerations regarding bedrock input in the calculations have also been provided.

Revised content is as follows:Based on the mean dynamic amplification factor spectra for four site categories, this study establishes the expression form of the design spectrum through numerical analysis of site models. Site effects constitute the core factor in defining design response spectrum curves, reflecting the alteration of spectral characteristics during seismic wave propagation from bedrock to ground surface due to soil filtering and resonance effects. Distinct site categories exhibit markedly different dynamic amplification properties: soft soil sites (Categories Ⅲ and Ⅳ) typically demonstrate longer characteristic periods and greater amplification of long-period components, whereas stiff sites (Categories Ⅰ and Ⅱ) exhibit short-period, high-amplitude amplification characteristics. Consequently, the modulating effect of site conditions on ground motion spectral shape must be fully accounted for during design response spectrum calibration.

Regarding the consideration of bedrock input, it is primarily applicable to the following scenarios: (1) When a definitive engineering bedrock surface exists beneath the project site with a bedrock wave velocity exceeding 500 m/s, the bedrock should be adopted as the reference plane for seismic motion input to accurately characterize the amplification effect of soil layers on seismic waves; (2) For sites with deep overburden deposits or complex soil layer structures, bedrock input is required to conduct one-dimensional or two-dimensional site seismic response analyses, thereby obtaining surface design ground motion parameters, followed by site adjustment through site classification. The Category Ⅰ–Ⅳ site records selected in this study all account for the influence of site conditions on ground motion, wherein Category Ⅰ sites approximate bedrock conditions, while Categories Ⅱ–Ⅳ sites exhibit varying degrees of soil amplification effects.

Response to Comment：Regarding the calibration error analysis, relevant reference cases have been cited for illustration. The error analysis Table 12 has been provided by calculating the standard deviation to demonstrate that the standard deviation is lower than that of the original version spectrum.

Revised content is as follows: Table 12 presents the error analysis for the two calibration methods. The calibration error analysis indicates that the standard deviation of the improved design response spectrum is significantly lower than that of the original design response spectrum, and its curve more accurately reflects the spectral characteristics of the seismic response spectrum. This result is consistent with Chopra (2012) regarding curved platform segments of design spectra providing better fitting to actual response spectra[44], and also aligns with the analysis results of Xie Lili et al. (2012) for Wenchuan earthquake records, thereby validating the importance of the improved spectral shape in engineering applications[45].

Conclusions

The conclusions commmonly describe the findings, the possible applications and future work. Two different sections should me used: Discussion and Conclusions.

Response to Comment：Dear r

Attachment Submitted filename: Response to Reviewers.docx

# Peer review: Decision Letter 1

**DOI:** 10.1371/journal.pone.0348599.r003

**Authors:** Alessandro Pluchino

20 Apr 2026

Statistical Shape Refinement and Genetic Algorithm Calibration of Design Response Spectra Based on Strong-Motion Records

PONE-D-26-02245R1

Dear Dr. Han,

We’re pleased to inform you that your manuscript has been judged scientifically suitable for publication and will be formally accepted for publication once it meets all outstanding technical requirements.

Within one week, you’ll receive an e-mail detailing the required amendments. When these have been addressed, you’ll receive a formal acceptance letter and your manuscript will be scheduled for publication.

An invoice will be generated when your article is formally accepted. Please note, if your institution has a publishing partnership with PLOS and your article meets the relevant criteria, all or part of your publication costs will be covered. Please make sure your user information is up-to-date by logging into Editorial Manager at Editorial Manager® and clicking the ‘Update My Information' link at the top of the page. For questions related to billing, please contact billing support.

If your institution or institutions have a press office, please notify them about your upcoming paper to help maximize its impact. If they’ll be preparing press materials, please inform our press team as soon as possible -- no later than 48 hours after receiving the formal acceptance. Your manuscript will remain under strict press embargo until 2 pm Eastern Time on the date of publication. For more information, please contact onepress@plos.org.

Kind regards,

Alessandro Pluchino

Academic Editor

PLOS One

Additional Editor Comments (optional):

Reviewers' comments:

Reviewer's Responses to Questions

**Comments to the Author**

1. If the authors have adequately addressed your comments raised in a previous round of review and you feel that this manuscript is now acceptable for publication, you may indicate that here to bypass the “Comments to the Author” section, enter your conflict of interest statement in the “Confidential to Editor” section, and submit your "Accept" recommendation.

Reviewer #1: All comments have been addressed

Reviewer #2: All comments have been addressed

**********

2. Is the manuscript technically sound, and do the data support the conclusions?

The manuscript must describe a technically sound piece of scientific research with data that supports the conclusions. Experiments must have been conducted rigorously, with appropriate controls, replication, and sample sizes. The conclusions must be drawn appropriately based on the data presented.

Reviewer #1: Yes

Reviewer #2: Yes

**********

3. Has the statistical analysis been performed appropriately and rigorously?

Reviewer #1: N/A

Reviewer #2: Yes

**********

4. Have the authors made all data underlying the findings in their manuscript fully available?

The PLOS Data policy requires authors to make all data underlying the findings described in their manuscript fully available without restriction, with rare exception (please refer to the Data Availability Statement in the manuscript PDF file). The data should be provided as part of the manuscript or its supporting information, or deposited to a public repository. For example, in addition to summary statistics, the data points behind means, medians and variance measures should be available. If there are restrictions on publicly sharing data—e.g. participant privacy or use of data from a third party—those must be specified.

Reviewer #1: Yes

Reviewer #2: Yes

**********

5. Is the manuscript presented in an intelligible fashion and written in standard English?

PLOS ONE does not copyedit accepted manuscripts, so the language in submitted articles must be clear, correct, and unambiguous. Any typographical or grammatical errors should be corrected at revision, so please note any specific errors here.

Reviewer #1: Yes

Reviewer #2: Yes

**********

6. Review Comments to the Author

Please use the space provided to explain your answers to the questions above. You may also include additional comments for the author, including concerns about dual publication, research ethics, or publication ethics. (Please upload your review as an attachment if it exceeds 20,000 characters)

Reviewer #1: The authors answered to all my requests and the paper has been developed and improved. It is ready for acceptance.

Reviewer #2: A genetic-algorithm-based calibration procedure was subsequently developed to determine optimal model parameters.

The proposed calibration framework offers a valuable reference for advancing design-response-spectrum studies and for the potential updating of seismic design codes.

My points have been addressed well and could be accepted.

**********

7. PLOS authors have the option to publish the peer review history of their article (what does this mean?). If published, this will include your full peer review and any attached files.

If you choose “no”, your identity will remain anonymous but your review may still be made public.

**Do you want your identity to be public for this peer review?**  For information about this choice, including consent withdrawal, please see our Privacy Policy.

Reviewer #1: No

Reviewer #2: No

**********

# Editor report: Acceptance letter

**DOI:** 10.1371/journal.pone.0348599.r004

**Authors:** Alessandro Pluchino

PONE-D-26-02245R1

PLOS One

Dear Dr. Han,

I'm pleased to inform you that your manuscript has been deemed suitable for publication in PLOS One. Congratulations! Your manuscript is now being handed over to our production team.

At this stage, our production department will prepare your paper for publication. This includes ensuring the following:

* All references, tables, and figures are properly cited

* All relevant supporting information is included in the manuscript submission,

* There are no issues that prevent the paper from being properly typeset

You will receive further instructions from the production team, including instructions on how to review your proof when it is ready. Please keep in mind that we are working through a large volume of accepted articles, so please give us a few days to review your paper and let you know the next and final steps.

Lastly, if your institution or institutions have a press office, please let them know about your upcoming paper now to help maximize its impact. If they'll be preparing press materials, please inform our press team within the next 48 hours. Your manuscript will remain under strict press embargo until 2 pm Eastern Time on the date of publication. For more information, please contact onepress@plos.org.

You will receive an invoice from PLOS for your publication fee after your manuscript has reached the completed accept phase. If you receive an email requesting payment before acceptance or for any other service, this may be a phishing scheme. Learn how to identify phishing emails and protect your accounts at https://explore.plos.org/phishing.

If we can help with anything else, please email us at customercare@plos.org.

Thank you for submitting your work to PLOS ONE and supporting open access.

Kind regards,

PLOS ONE Editorial Office Staff

on behalf of

Dr. Alessandro Pluchino

Academic Editor

PLOS One
